# Interrelationships between diet quality and health-related quality of life in Irish adults living with cystic fibrosis

**DOI:** 10.1007/s00394-025-03766-y

**Published:** 2025-07-24

**Authors:** Cian Greaney, Ellen McCarthy, Lauren O’Brien, Sarah Tecklenborg, Ciara Howlett, Karen Cronin, Clodagh Landers, Mary Connolly, Derbhla O’Sullivan, Aoife Whiston, Katie Robinson, Audrey Tierney

**Affiliations:** 1 School of Allied Health, University of Limerick, Limerick, Ireland; 2 Health Research Institute, University of Limerick, Limerick, Ireland; 3 Cystic Fibrosis Ireland, Rathmines, Dublin, Ireland; 4 Cork University Hospital, Cork, Ireland; 5 St. Vincent’s University Hospital, Dublin 4, Dublin, Ireland; 6 University Hospital Galway, Galway, Ireland; 7 University Hospital Limerick, Dooradoyle, Limerick, Ireland; 8 Department of Psychology, University of Limerick, Limerick, Ireland; 9 Aging Research Centre, Health Research Institute, University of Limerick, Limerick, Ireland; 10 Centre for Implementation Research, Health Research Institute, University of Limerick, Limerick, Ireland; 11 Discipline of Food, Nutrition and Dietetics, La Trobe University, Melbourne, VIC 3086 Australia

**Keywords:** Cystic fibrosis, Diet quality, Patient-reported outcomes, Quality of life, Symptoms

## Abstract

**Purpose:**

In the Cystic Fibrosis (CF) modulator era, focus for many has shifted from growth and survival to prevention of diet-related chronic diseases. In doing so, diet quality should be prioritised. However, factors influencing diet quality in CF have yet to be examined. This study aims to explore relationships between health-related quality of life (HRQoL), demographic and clinical characteristics, and diet quality in adults living with CF.

**Methods:**

Cross-sectional study of Irish adults with CF. Dietary intake was assessed using three-day food diaries and analysed using the Healthy Eating Index-2020 (HEI-2020) and Diet Quality Index-International (DQI-I), both scored 0–100, with higher scores indicating better diet quality. Patient-reported outcome measure questionnaires collected HRQoL data and symptom status. Data was statistically analysed in SPSS®.

**Results:**

Among *N* = 73 participants (female: 56.2%, age: 35.1 ± 10.3 years), 79.4% were taking CFTR modulators. Mean predicted percentage forced expiratory volume (FEV_1_%) was 78.7 ± 24.9%, and median body mass index was 23.9 (4.2) kg/m^2^. Mean HEI-2020 and DQI-I scores were 59.3 ± 12.4 and 51.2 ± 9.8, respectively. Anxiety/depression symptoms were reported by 43.1% of participants, and 30.6% reported the presence of pain/discomfort. Bloating in stomach was reported in 68.1% of participants, with 43.1% reporting moderate to severe symptoms. FEV_1_% and digestive symptoms were independently positive significant predictors of HEI-2020 scores (R^2^adj = 0.166, *n* = 67, *p* = 0.004).

**Conclusion:**

In the modulator era, suboptimal vitality, anxiety / depression, and gut symptoms remain prevalent among adults living with CF. Future dietary interventions should aim to improve diet quality and consider incorporating dietary patterns that positively influence the gut microbiome.

**Supplementary Information:**

The online version contains supplementary material available at 10.1007/s00394-025-03766-y.

## Introduction

Cystic Fibrosis (CF) is a multiorgan disorder primarily characterised by recurrent lung infections which can lead to poorer pulmonary function and a shorter lifespan [1]. In recent years, improvements in clinical practices and variant specific therapies like CF transmembrane conductance regulator (CFTR) modulator therapy have altered the trajectory of CF [2], with many people living with CF (PwCF) experiencing better health outcomes and subsequentially living to an older age [3].

Weight gain and body composition changes are reported with the use of variant specific therapies [4–6], and while much of this weight gain is positive, progressing from underweight to normal weight body mass index (BMI) ranges, rises in incidences of overweight / obese BMI classifications (≥ 25 kg/m^2^) in adult PwCF have been reported in both retrospective [7] and prospective [8] studies. Furthermore, diabetes and cardiovascular disease are now established concerns in CF. A longitudinal study (*N* = 40,096) evaluating European CF registry data reported increases in CF-related diabetes incidences with age (< 10 years: 0.8%; 10–19 years: 9.7%; 20–29 years: 24.1%; ≥ 30 years: 32.7%) [9], with higher mortality rates [10] and poorer cardiovascular health outcomes commonly presented in diabetic PwCF [11].

A nutrient dense, high quality diet typically represents diets associated with lower risk of developing diet-related chronic diseases (i.e., obesity, type-II diabetes, cardiovascular disease) [12], with diet quality referring to a pattern of eating or a display of heterogeneity spanning key food groups outlined in nutrition guidelines [13]. The latest CF European Nutrition Guidelines have alluded to the importance of diet quality amongst PwCF [1]. Various index tools for measuring diet quality have been developed and validated and although scarcely applied within CF settings, have revealed suboptimal diet quality amongst PwCF [14, 15]. A lack of heterogeneity exists in methods used to assess diet quality in CF [14]. With diet quality indices providing a means of assessing and mapping diet quality to objective measures, it is necessary to evaluate what index may be the most appropriate to adopt and whether findings are replicable between different diet quality indices in a CF setting.

Diet quality has been associated with both quality of life domains and objective measures of health (predicted percentage forced expiratory volume (FEV_1_%), inflammatory markers and metabolic disease risk factors) in the general population [16–19], the combination of which is often termed health-related quality of life (HRQoL) [20]. Patient-reported outcome measures (PROMs) can yield important information about patients’ perspectives on the impact of treatment on HRQoL, serving as valuable indicators for the benefit of interventions [21] with their use widely supported and applied in CF research [22]. The US Food and Drug Administration supports the use of HRQoL measures as clinical trial outcomes [23], thus, identifying the interrelationships between diet quality and HRQoL and addressing these through interventions could offer an avenue for reducing and preventing the onset of diet-related chronic diseases in CF.

While the importance of diet quality in managing long-term health outcomes in PwCF has been acknowledged in recent guidelines [1], little is known about how diet quality is influenced by broader aspects of HRQoL in this population. To date, none of the studies identified as measuring diet quality [24–27] in a systematic review on dietary intakes and diet quality in adults living with CF [14], have examined the interrelationships between diet quality and HRQOL, highlighting a critical gap in the existing literature. Given the prevailing relationship between diet quality and chronic diseases, it is crucial to assess the factors associated with diet quality in PwCF to inform future dietary and lifestyle interventions, clinical practices and nutrition guidelines. Considering the known adverse health outcomes associated with poor diet quality and the paucity of research to date exploring interrelationships between diet quality and HRQoL in PwCF, this study will evaluate HRQoL (via PROM questionnaires) and explore associations between PROMs and diet quality, and demographic, clinical and PROM predictors of diet quality. A secondary objective will be to assess whether similar findings regarding diet quality are observed, despite the use of different diet quality indices within the CF context.

## Methods

### Study design

An observational, cross-sectional study was completed adhering to STROBE guidelines [28] with a study protocol previously published [29]. Quantitative data were gathered using a demographic and self-reported health questionnaire, three-day food diary and PROM questionnaires which included the CF Questionnaire– Revised (CFQ-R) (teen / adult version), EuroQol 5-Dimension 5-Level questionnaire (EQ-5D-5L), Patient Assessment of Gastrointestinal Symptoms (PAGI-SYM) and Patient Assessment of Constipation Symptoms (PAC-SYM) questionnaires to capture a wide range of HRQoL measures. Study material was offered in digital and paper formats. The Electronic Data Capture system (EDC), Castor, Version 1.6 (Ciwit B.V., The Netherlands) collected and managed questionnaire data. The smartphone application, Libro, from the dietary analysis software, Nutritics, Version 5.7 Research Edition (Nutritics Ltd.: Dublin, Ireland) [30] was used to gather three-day food diary data digitally. Estimating portion sizes through common household measures and documentation of cooking methods were required within food diaries. Data were exported and analysed through Nutritics, generating comprehensive daily and mean energy, macronutrient, and micronutrient intake values using Nutritics, Ireland (2009 Irish Food Composition Database). The full recruitment strategy and dietary intake assessment has been previously published [15].

### Collecting and measuring PROMs

Once consent was obtained, participants received a link via email to access the PROM, demographic and self-reported health questionnaires on Castor EDC. Data was manually transferred from Castor EDC into Microsoft Excel in preparation for statistical analysis.

#### CFQ-R

A 50-item self-administered, validated and disease-specific instrument incorporating general and CF-specific domains of HRQoL [31] was completed by participants. Item responses were collected via four-point Likert scale scores and standardised across scales to range from 0 to 100, with greater scores demonstrating better self-reports of HRQoL. Items in each domain were averaged to produce final domain scores for individuals, with higher scores representing better self-reports of HRQoL for each domain [31].

#### EQ-5D-5L

The EQ-5D-5L is a widely accepted universal self-report measure for assessing utilities, yielding a single index value of health status [32]. Five dimensions of general health status were assessed via five-level Likert scales which measure levels of severity (no, slight, moderate, severe, and extreme problems). Self-rated health was assessed using the EuroQol Visual Analogue Scale where health is rated from 0 (worst imaginable health) to 100 (best imaginable health). Responses to the five-dimensions were quantified from 1 to 5 following the severity pattern described. Individual responses to each dimension were then collated to provide a five-figure score, with ‘11111’ representing no difficulty with any level, and ‘55555’ representing extreme difficulty with all levels [32]. These profiles were entered into the EQ-5D-5L Index Value Calculator to map results to an Irish value set [33].

#### PAGI-SYM

The PAGI-SYM is a validated, self-reported measure of upper gastrointestinal symptom severity (0–5: 0 = no symptoms; 5 = very severe symptoms) with 20-items scored across six subscales [34]. The 20 responses were averaged for each participant to produce a total score. Items within each subscale were averaged to produce final subscale scores.

#### PAC-SYM

The PAC-SYM is a 12-item validated self-report tool with three subscales used to evaluate constipation symptom severity. Each item can be scored from zero (no symptom) to four (very severe symptoms) [35]. The 12 responses were averaged for each participant to produce a final PAC-SYM score, and items of each of the three subscales were averaged to produce a final subscale score. Both questionnaires rely on a 2-week recall period.

### Measuring diet quality

Diet quality was assessed via the Healthy Eating Index– 2020 (HEI-2020) as previously described [15]. Although developed for the American context [36], the HEI-2020 is a valuable tool for assessing diet quality and with recent European CF guidelines recommending general population dietary practices [1], the principles of the HEI-2020 mirror Irish population nutrition guidelines, promoting fruits, vegetables, whole grains, and lean proteins and limiting intakes of saturated fat, sugar and salt [37]. The HEI-2020 has also been previously applied in CF contexts [25, 38], and used to evaluate diet-health relationships in an Irish contexts [39, 40]. Additionally, diet quality was also assessed via the Diet Quality Index– International (DQI-I). Final scores for both indices range from 0 to 100 where higher scores reflect better diet quality [36, 41]. While no previous research has reported a DQI-I score for PwCF, DQI-I can pinpoint exact nutritional deficiencies typically less prevalent in developed countries [42] that can present in CF [43], alongside matching developed country dietary concerns. The DQI-I also accommodates both overnutrition and undernutrition [42], relevant in the modulator era of CF where a paradigm shift has occurred in nutrition status according to registry data [44].

### Statistical analysis

Statistical analysis was conducted with SPSS^®^ Statistics for Windows, Version 29 (IBM Corp., Released 2022). Descriptive statistics were used to describe baseline characteristics of the cohort. Distribution of data was tested using the Kolmogorov Smirnov test of normality, where a significance of *p* > 0.05 identified data as normally distributed. Data were presented as means ± standard deviations, median (interquartile range) and frequencies (*n* and %) where appropriate. Where variables were not normally distributed, variables were noted with an asterisk. To assess univariate differences in PROMs with diet quality, HEI-2020 and DQI-I diet quality scores were categorised into above or below the mean value for each diet quality score. Independent t-tests (non-parametric: Mann Whitney-U test) compared continuous variables between groupings of two categories (e.g., above / below mean diet quality scores, differences between genders, etc.). One-way ANOVA (non-parametric: Kruskal–Wallis) tests were applied to compare continuous variables between participant groupings of three or more categories (e.g., income status) and post hoc Bonferroni tests were performed to assess differences between specific categories. Pearson's Chi-square was used to test differences between categorical variables. Total diet quality scores and scores for each component of the score were calculated and compared between demographic groups using appropriate statistical tests as previously described. Pearson’s correlation (non-parametric: Spearman’s rank correlation coefficient) was used to explore associations between scale data (e.g., diet quality index scores, CFQ-R domain scores, etc.). Multiple (linear) regression models were performed to assess what variables most strongly predict an increase in diet quality scores using an exploratory approach. Collinearity was addressed by swapping variables with strong (*R* ≥ 0.7) and significant (*p* < 0.05) Pearson’s correlations between models. A formal a priori power analysis was not conducted, as this study was exploratory in nature and aimed to identify potential associations between demographic, clinical, and PROM variables and diet quality scores. The number of variables used within reported linear regression models were determined based on the sample size, with one variable per 15 participants typically recommended [45]. Variables identified as significantly correlated with diet quality scores were considered for inclusion in the final presented model. Larger linear regression models containing all demographic, clinical and PROM variables were completed only to cross-check results of linear regression models with the number of variables appropriate for the sample size. Variables regularly significant across multiple linear regression models were included in the final reported linear regression model, alongside contextually important variables. Correction for multiple comparisons was not applied, as predictors were selected based on theoretical relevance and consistency across models, rather than independent hypothesis testing.

## Results

The demographic and clinical characteristics of participants are displayed in Table 1. A total of *N* = 73 participants took part in the study (mean age: 35.1 ± 10.3 years; female: 56.2%), with a median BMI of 23.9 ± 4.2 kg/m^2^ and mean FEV_1_% of 78.7 ± 24.9% reported, and 79.4% reported to be taking modulators. Demographic and clinical characteristics, dietary intakes and diet quality data on *n* = 68 of the cohort, have been previously published [15]. All participants completed the CFQ-R, while *n* = 1 participant did not complete the EQ-5D-5L, and *n* = 3 participants did not complete the PAGI-SYM. Of those who did not complete PAGI-SYM, *n* = 1 did not complete the PAC-SYM, and *n* = 1 returned the PAC-SYM partially completed.

**Table 1:** Demographic and clinical characteristics of Irish adults living with CF who participated in this cross-sectional study

	All (*N* = 73)	Male (*n* = 32)	Female (*n* = 41)	*p*-value
Age (y) (range: 19–68)	35.1 ± 10.3	37.3 ± 10.7	33.5 ± 9.7	0.565
BMI (kg/m^2^)*	23.9 (4.2)	25.4 (3.4)	22.3 (3.2)	< 0.001
*BMI Classification n(%)*^~^				0.009
Underweight (< 20)	1 (1.4)	0 (0.0)	1 (2.4)	–
Normal (20–24.9)	44 (60.3)	13 (40.6)	31 (75.6)	–
Overweight (25–29.9)	24 (32.9)	17 (53.1)	7 (17.1)	–
Obese (≥ 30)	4 (5.5)	2 (6.3)	2 (4.9)	–
	*n* = 72		*n* = 40	
FEV_1_%	78.7 ± 24.9	78.8 ± 26.2	78.6 ± 24.2	0.390
*FEV*_1_*% Categories n(%)*				0.975
< 40%	6 (8.3)	3 (9.4)	3 (7.5)	–
40–69.99%	17 (23.6)	8 (25.0)	9 (22.5)	–
70–79.99%	10 (13.9)	4 (12.5)	6 (15.0)	–
≥ 80%	39 (54.2)	17 (53.1)	22 (55.0)	–
*Hospital Admission*				–
Since last admission (days)*	815.0 (1094.8)	893.0 (1030.5)	742.5 (1085.5)	0.297
Last admission length (days)*	13.5 (9.0)	13.0 (9.0)	13.5 (9.5)	0.818
No admissions in last year *n*(%)	52 (72.2)	26 (81.3)	26 (65.0)	0.126
*Highest Education n(%)*				0.650
Secondary level	11 (15.3)	5 (15.6)	6 (15.0)	–
Tertiary level	40 (55.6)	16 (50.0)	24 (60.0)	–
Postgraduate level	21 (29.2)	11 (34.4)	10 (25.0)	–
*Income Status n(%)*				< 0.001
Full income	33 (45.8)	22 (68.8)	11 (27.5)	–
Part–time income	14 (19.4)	4 (12.5)	10 (25.0)	–
No work–based income	25 (24.7)	6 (18.8)	19 (47.5)	–
*Area of Living n(%)*				0.096
City	28 (38.9)	16 (50.0)	12 (30.0)	–
Town	12 (16.7)	7 (21.9)	5 (12.5)	–
Village	9 (12.5)	3 (9.4)	6 (15.0)	–
Countryside	23 (31.9)	6 (18.8)	17 (42.5)	–
*Living Situation n(%)*				0.534
Living alone	7 (9.7)	4 (12.5)	3 (7.5)	–
Living with housemates	15 (20.8)	5 (15.6)	10 (25.0)	–
Living with family/relatives	50 (69.4)	23 (71.9)	27 (67.5)	–
*Clinical Characteristics n(%)*				
Pancreatic insufficiency	51 (70.8)	24 (75.0)	27 (67.5)	0.487
CF-related diabetes	19 (26.4)	9 (28.1)	10 (25.0)	0.765
GORD	16 (22.2)	7 (21.9)	9 (22.5)	0.949
CF-related liver disease	8 (11.1)	6 (18.8)	2 (5.0)	0.065
CF-related bone disease	6 (8.3)	1 (3.1)	5 (12.5)	0.153
*Medication Use n(%)*				
Modulators	57 (79.2)	22 (68.8)	35 (87.5)	0.052
Antibiotics	37 (51.4)	18 (56.3)	19 (47.5)	0.460
Mucolytics	13 (18.1)	6 (18.8)	7 (17.5)	0.891
Bronchodilators	27 (37.5)	12 (37.5)	15 (37.5)	1.000
Insulin	19 (26.4)	9 (28.1)	10 (25.0)	0.765
Steroids	10 (13.9)	7 (21.9)	3 (7.5)	0.080
*Supplement Use n(%)*				
Fat-soluble vitamins	61 (84.7)	25 (78.1)	36 (90.0)	0.164
Nutritional supplement drinks	10 (13.9)	6 (18.8)	4 (10.0)	0.286

Parametric values are presented as mean ± SD and non-parametric values as median (interquartile range)

*P*-values were derived with chi-square tests, independent sample t-tests and ANOVA tests (Non-parametric: Mann Whitney U and Kruskal–Wallis tests)

^*^Non-parametric test

^**~**^World Health Organisation cut-off values [46]

Significance derived from a *p*-value of < 0.05

BMI, body mass index; FEV_1_%, percentage of predicted force expiratory volume for age, sex, ethnicity, and height; GORD, gastro-oesophageal reflux disease; SD, standard deviation

### HRQoL and symptom severity in adult PwCF

**Fig. 1:**
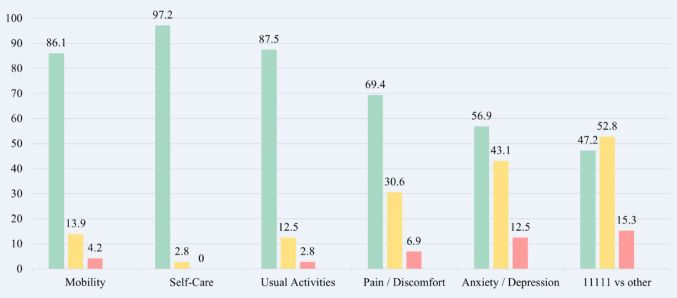
Frequency (%) of utility problem / symptom severity in adults living with CF measured by the EQ-5D-5L. The green bars represent the percentage of individuals reporting no problems / symptoms (1), the yellow bars represent the percentage of individuals reporting a problem / symptom present (2-4), the pink bars represent the percentage of individuals reporting moderate to severe problems / symptoms (3-4), and the blue bars represent the percentage of individuals reporting extreme problems /symptoms (5)

**Fig. 2:**
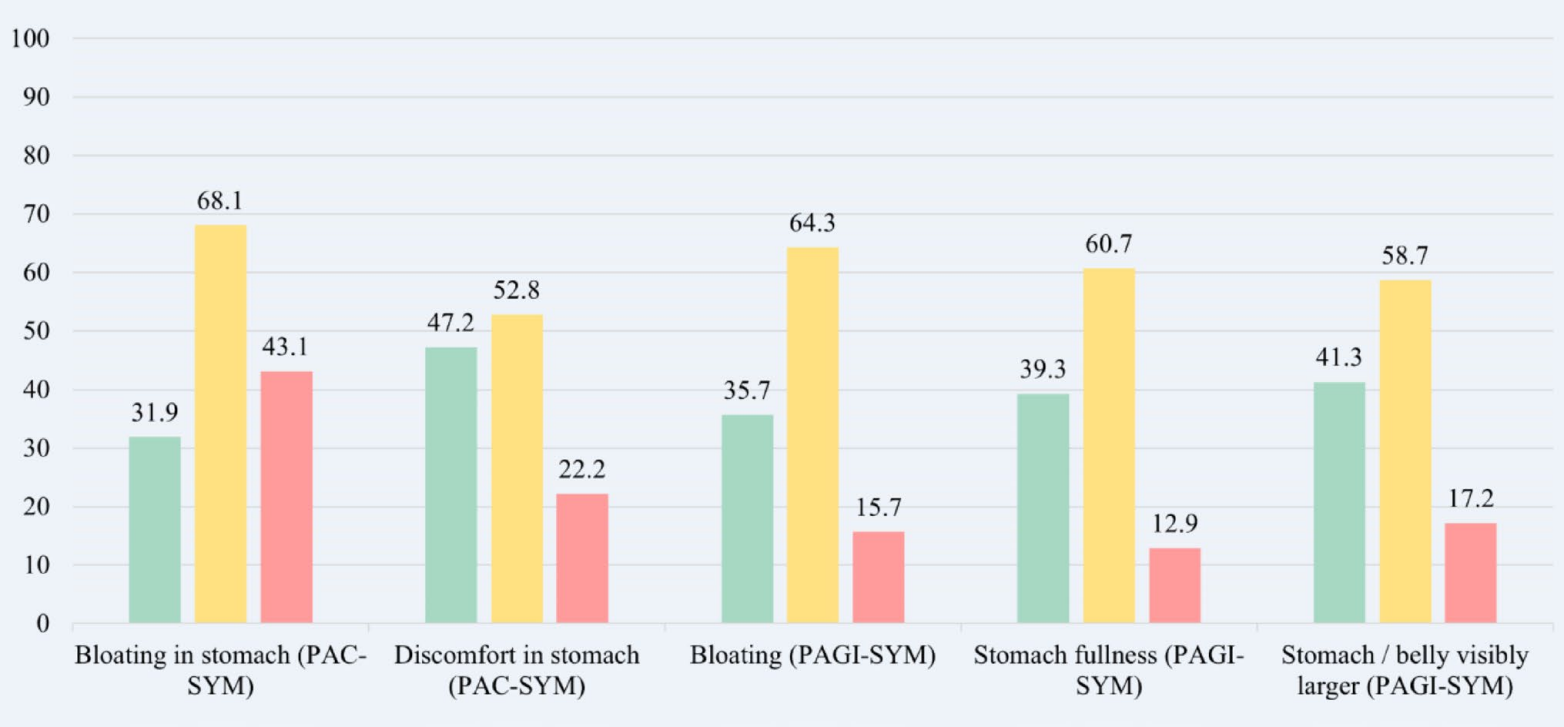
Most common gastrointestinal symptoms reported (%) and gut symptom severity in adults living with CF measured by PAC- / PAGI-SYM. The green bars represent the percentage of individuals reporting no symptoms, the yellow bars represent the percentage of individuals reporting symptoms present, and the pink bars represent the percentage of individuals reporting moderate to very severe symptoms

A total of 43.1% of participants reported slight to severe symptoms of anxiety / depression (EQ-5D-5L) and 30.6% reported experiencing symptoms of pain / discomfort, with no report of extreme problems / symptoms in any dimension of the EQ-5D-5L. Furthermore, 47.2% of participants reported no problem / symptom across any dimension of the EQ-5D-5L (11111), with 52.8% experiencing a problem / symptom with at least one dimension (other) (Fig. 1). The most commonly reported gastrointestinal symptom was bloating in the stomach (PAC-SYM) (68.1%), with 43.1% reporting moderate to very severe symptoms [2–4] (Fig. 2). The highest scoring CFQ-R domains were eating problems and weight with vitality being the lowest scoring domain (Table 2). A subgroup analysis of PROMs is presented in Supplementary Material 1.

**Table 2:** Differences in PROMs between participants above or below the mean HEI-2020 and DQI-I scores

	Below Mean HEI-2020 Score (*n* = 32)	Above Mean HEI-2020 Score (*n* = 36)	*p*-value (HEI-2020)	Below Mean DQI-I Score (*n* = 35)	Above Mean DQI-I Score (*n* = 33)	*p*-value (DQI-I)
CFQ-R Domain Scores (0–100)						
Physical Functioning	85.4 (45.8)	95.8 (29.2)	0.055	87.5 (33.3)	95.8 (29.2)	0.448
Vitality	66.7 (25.0)	66.7 (25.0)	0.686	66.7 (25.0)	66.7 (25.0)	0.490
Emotional Functioning	73.3 (13.3)	80.0 (18.3)	0.184	73.3 (26.7)	80.0 (16.7)	0.215
Eating Problems	100.0 (11.1)	100.0 (11.1)	0.777	100.0 (11.1)	100.0 (11.1)	0.541
Treatment Burden	72.2 (33.3)	77.8 (33.3)	0.480	77.8 (33.3)	66.7 (33.3)	0.694
Health Perceptions	77.8 (30.6)	77.8 (30.6)	0.237	77.8 (22.2)	77.8 (33.3)	0.591
Social / School Functioning	66.7 (37.5)	83.3 (27.8)	0.059	66.7 (38.9)	77.8 (30.6)	0.364
Body Image	77.8 (44.5)	83.3 (41.7)	0.621	77.8 (33.3)	88.9 (38.9)	0.112
Role Functioning	91.7 (16.7)	91.7 (16.7)	0.448	91.7 (16.7)	91.7 (16.7)	0.714
Weight	100.0 (0.0)	100.0 (0.0)	0.862	100.0 (0.0)	100.0 (16.7)	0.590
Respiratory Symptoms	91.7 (11.1)	91.7 (16.7)	0.688	88.9 (11.1)	94.4 (16.7)	0.183
Digestive Symptoms	77.8 (38.9)	88.9 (33.3)	0.216	77.8 (44.5)	88.9 (33.3)	0.486
		*n* = 35		*n* = 34		
EQ-5D-5L Total Scores			0.144			0.051
11111 *n*(%)	12 (37.5)	20 (57.1)		12 (35.3)	20 (60.6)	
any other health state *n*(%)	20 (62.5)	15 (42.9)		22 (64.7)	13 (39.4)	
EQ Visual Analogue Scale score (0–100)	80.5 (20.8)	85.0 (10.0)	0.192	82.0 (16.3)	85 (15.0)	0.821
*Having any problem / symptom in five dimensions (score* ≥ *2) n(%)*						
Mobility	8 (25.0)	2 (5.7)	0.039	5 (14.7)	5 (15.2)	1.000
Self-care	2 (6.3)	0 (0.0)	0.224	2 (5.9)	0 (0.0)	0.493
Usual Activities*	5 (15.6)	4 (11.4)	0.727	2 (5.9)	7 (21.2)	0.083
Pain / Discomfort	13 (40.6)	7 (20.0)	0.108	14 (41.2)	6 (18.2)	0.061
Anxiety / Depression	15 (46.9)	13 (37.1)	0.288	17 (50.0)	11 (33.3)	0.218
*Dimension scores (1–5)*						
Mobility	1.0 (0.8)	1.0 (0.0)	0.024	1.0 (0.0)	1.0 (0.0)	0.992
Self-care	1.0 (0.0)	1.0 (0.0)	0.136	1.0 (0.0)	1.0 (0.0)	0.160
Usual Activities^~^	1.0 (0.0)	1.0 (0.0)	0.560	1.0 (0.0)	1.0 (0.0)	0.079
Pain / Discomfort	1.0 (1.0)	1.0 (0.0)	0.074	1.0 (1.0)	1.0 (0.0)	0.077
Anxiety / Depression	1.0 (1.0)	1.0 (1.0)	0.347	1.5 (1.0)	1.0 (1.0)	0.115
				*n* = 35	*n* = 32	
PAC-SYM Score (0–4)	0.4 (0.7)	0.3 (0.5)	0.347	0.4 (0.7)	0.3 (0.6)	0.430
*Subscales*						
Abdominal Symptoms	0.6 (1.0)	0.5 (1.0)	0.196	0.5 (1.0)	0.5 (0.9)	0.445
Rectal Symptoms	0.0 (0.3)	0.0 (0.0)	0.388	0.0 (0.3)	0.0 (0.0)	0.402
Stool Symptoms	0.2 (0.6)	0.2 (0.8)	0.968	0.4 (0.6)	0.0 (0.6)	0.156
*Constipation symptom details*						
discomfort in stomach	1.0 (1.0)	0.0 (1.0)	0.629	0.0 (1.0)	1.0 (1.0)	0.629
pain in your stomach	0.0 (1.0)	0.0 (1.0)	0.255	0.0 (1.0)	0.0 (1.0)	0.728
bloating in your stomach	1.0 (1.8)	1.0 (2.0)	0.239	1.0 (2.0)	1.0 (2.0)	0.129
stomach cramps	0.0 (1.0)	0.0 (0.0)	0.120	0.0 (1.0)	0.0 (0.0)	0.188
painful bowel movements	0.0 (0.0)	0.0 (0.0)	0.628	0.0 (0.0)	0.0 (0.0)	0.471
rectal burning during / after a bowel movement	0.0 (0.0)	0.0 (0.0)	0.611	0.0 (0.0)	0.0 (0.0)	0.303
rectal bleeding or tearing during / after a bowel movement	0.0 (0.0)	0.0 (0.0)	0.394	0.0 (0.0)	0.0 (0.0)	0.641
incomplete bowel movement, felt like you didn't finish	0.0 (1.0)	0.0 (1.0)	0.867	1.0 (1.0)	0.0 (1.0)	0.269
bowel movements were too hard	0.0 (0.0)	0.0 (0.0)	0.877	0.0 (0.0)	0.0 (0.0)	0.443
bowel movements were too small	0.0 (1.0)	0.0 (1.0)	0.956	0.0 (1.0)	0.0 (1.0)	0.702
straining or squeezing to try to pass bowel movements	0.0 (1.0)	0.0 (1.0)	0.620	0.0 (1.0)	0.0 (0.0)	0.081
feeling like you had to pass a bowel movement but you could not ('false alarm')	0.0 (0.0)	0.0 (0.0)	0.378	0.0 (0.0)	0.0 (0.0)	0.909
		*n* = 34			*n* = 31	
PAGI-SYM Score (0–5)	0.4 (0.6)	0.2 (0.6)	0.070	0.4 (0.6)	0.2 (0.5)	0.044
*Subscales*						
Heartburn / Regurgitation	0.1 (0.5)	0.0 (0.3)	0.359	0.1 (0.6)	0.0 (0.3)	0.407
Nausea / Vomiting	0.0 (0.3)	0.0 (0.1)	0.402	0.0 (0.3)	0.0 (0.0)	0.056
Fullness / Satiety	0.6 (0.8)	0.1 (0.8)	0.040	0.8 (0.8)	0.0 (0.5)	0.033
Bloating	1.0 (1.5)	0.5 (1.6)	0.077	1.0 (2.0)	0.5 (1.0)	0.022
Upper-Abdominal Pain	0.0 (0.5)	0.0 (0.0)	0.437	0.0 (0.0)	0.0 (0.5)	0.857
Lower-Abdominal Pain	0.0 (0.5)	0.0 (0.5)	0.878	0.0 (0.5)	0.0 (0.5)	0.933
*Gastrointestinal symptom details*						
Nausea	0.0 (1.0)	0.0 (0.0)	0.257	0.0 (1.0)	0.0 (0.0)	0.103
Retching	0.0 (0.0)	0.0 (0.0)	0.654	0.0 (0.0)	0.0 (0.0)	0.228
Vomiting	0.0 (0.0)	0.0 (0.0)	0.594	0.0 (0.0)	0.0 (0.0)	0.631
Stomach fullness	1.0 (2.0)	0.5 (2.0)	0.092	1.0 (2.0)	0.0 (1.0)	0.029
Not able to finish a normal sized meal	0.0 (1.0)	0.0 (0.0)	0.139	0.0 (1.0)	0.0 (0.0)	0.769
Feeling excessively full after meals	0.0 (1.8)	0.0 (1.0)	0.185	0.0 (2.0)	0.0 (1.0)	0.225
Loss of appetite	0.0 (0.8)	0.0 (0.3)	0.986	0.0 (1.0)	0.0 (0.0)	0.878
Bloating	1.0 (2.0)	1.0 (2.0)	0.172	1.0 (2.0)	0.0 (1.0)	0.043
Stomach or belly visibly larger	1.0 (2.0)	0.0 (1.3)	0.082	1.0 (2.0)	0.0 (1.0)	0.020
Upper abdominal pain	0.0 (0.0)	0.0 (0.0)	0.690	0.0 (0.0)	0.0 (0.0)	0.643
Upper abdominal discomfort	0.0 (0.8)	0.0 (0.0)	0.456	0.0 (0.0)	0.0 (0.0)	0.871
Lower abdominal pain	0.0 (0.0)	0.0 (0.0)	0.692	0.0 (0.0)	0.0 (0.0)	0.851
Lower abdominal discomfort	0.0 (1.0)	0.0 (1.0)	0.950	0.0 (1.0)	0.0 (1.0)	0.673
Heartburn during the day	0.0 (0.0)	0.0 (1.0)	0.758	0.0 (1.0)	0.0 (0.0)	0.751
Heartburn when lying down	0.0 (1.0)	0.0 (0.0)	0.136	0.0 (1.0)	0.0 (0.0)	0.097
Discomfort inside your chest during the day	0.0 (0.0)	0.0 (0.0)	0.560	0.0 (0.0)	0.0 (0.0)	0.833
Discomfort inside your chest at night	0.0 (0.0)	0.0 (0.0)	0.456	0.0 (0.0)	0.0 (0.0)	0.837
Regurgitation or reflux during the day	0.0 (1.0)	0.0 (0.0)	0.365	0.0 (1.0)	0.0 (0.0)	0.751
Regurgitation or reflux when lying down	0.0 (1.0)	0.0 (0.0)	0.042	0.0 (0.0)	0.0 (0.0)	0.702
Bitter, acid or sour taste in your mouth	0.0 (0.0)	0.0 (0.0)	0.484	0.0 (0.0)	0.0 (0.0)	0.298

Values are represented as median (interquartile range) or *n*(%)

*P*-values were derived with chi-square tests, Mann Whitney U and Kruskal–Wallis tests (non-parametric)

Significance derived from a *p*-value of < 0.05

^*^Work, study, housework, family or leisure activities

HEI-2020, Healthy Eating Index– 2020; DQI-I, Diet Quality Index– International; CFQ-R, Cystic Fibrosis Questionnaire– Revised; EQ-5D-5L, EuroQol 5-dimension 5-level questionnaire; EQ, EuroQol; PAC-SYM, Patient Assessment of Constipation Symptoms questionnaire; PAGI-SYM, Patient Assessment of Upper Gastrointestinal Symptoms questionnaire

### Interrelationships between PROMs and diet quality scores

#### Differences in PROMs in adult PwCF by diet quality scores.

As previously reported [15], the mean HEI-2020 score was 59.3 ± 12.4. The mean DQI-I score was 51.2 ± 9.8. DQI-I component scores and subgroup analysis are presented in Supplementary Material 2. Participants scoring below the mean HEI-2020 score (i.e., lower diet quality) reported having significantly more problems with mobility (EQ-5D-5L mobility score ≥ 2) (*p* = 0.039), producing a significantly higher median EQ-5D-5L mobility dimension score (*p* = 0.024), alongside producing a significantly higher median fullness / satiety PAGI-SYM subscale score (*p* = 0.040) than participants above the mean HEI-2020 score. Participants scoring below the mean DQI-I score reported a significantly higher aggregated PAGI-SYM score (*p* = 0.044), alongside PAGI-SYM subscales of fullness / satiety (*p* = 0.033) and bloating (*p* = 0.022) compared to participants above the mean DQI-I score with no other significant differences observed (Table 2).

#### HEI-2020 demographic and clinical predictors

Pearson’s correlations revealed that HEI-2020 and DQI-I diet quality scores were significantly, moderately and positively correlated (*r* = 0.515,* p* = 0.000) and FEV_1_% was the only demographic or clinical variable significantly and moderately correlated with HEI-2020 scores (*r* = 0.332,* p* = 0.006). Multiple linear regressions were performed using four independent variables based on the sample size of* n* = 68 (larger linear regression model examples presented in Supplementary Material 3). Demographic and clinical variable linear regression models evaluating predictors of HEI-2020 revealed FEV_1_% as an independently positive significant predictor (*p* = 0.008), with the model (*p* = 0.005) accounting for 15.9% (R^2^adj = 0.159) of variance in HEI-2020 scores (Table 3).

**Table 3:** Regression 1: Pearson’s correlations amongst the dependent (HEI-2020 score) and independent variables and coefficients for predicting HEI-2020 scores in adults living with CF

Regression 1 (Demographics)	1	2	3	4	5
1. HEI-2020 Score (0–100)	–	−0.134	0.332**	−0.279*	−0.208*
2. Sex / Gender (male / female)		–	0.031	0.196	−0.092
3. FEV_1_%			–	−0.127	0.003
4. Modulator Use (yes / no)				–	0.189
5. Pancreatic Insufficiency (yes / no)					–

Variable	*B*	95% CI	β	*t*	*p*
Constant (HEI-2020 Score)	57.897	[45.467, 70.227]	–	9.387	< 0.001
Sex/Gender (male / female)	−3.185	[−9.052, 2.683]	−0.126	−1.085	0.282
FEV_1_%	0.157	[0.043, 0.271]	0.314	2.752	0.008
Modulator Use (yes / no)	−5.626	[−13.107, 1.855]	−0.179	−1.503	0.138
Pancreatic Insufficiency (yes / no)	−5.572	[−12.496, 1.352]	−0.187	−1.609	0.113

^*^p < 0.05. **p < 0.01.* n* = 67

R^2^adj = 0.159 (*n* = 67, *p* = 0.005)

Abbreviations: HEI-2020, Healthy Eating Index 2020; FEV_1_%, predicted percentage forced expiratory volume; CI, confidence interval of *B*

#### DQI-I demographic and clinical predictors

Pearson’s correlations revealed that no demographic or clinical variable was significantly and moderately correlated with DQI-I scores. Linear regression models evaluating predictors of DQI-I were not significant (*p* = 0.065). However, FEV_1_% was an independently positive significant variable within the model (*p* = 0.023) (Table 4).

**Table 4:** Regression 2: Pearsons correlations amongst the dependent (DQI-I score) and independent variables and coefficients for predicting DQI-I scores in adults living with CF

Regression 2 (Demographics)	1	2	3	4	5
1. DQI-I Score (0–100)	–	−0.151	0.143	0.232**	−0.148
2. Sex / Gender (male / female)		–	–0.187	0.031	0.196
3. Age (years)			–	−0.317**	0.083
4. FEV_1_%				–	−0.127
5. Modulator use (yes / no)					–

Variable	*B*	95% CI	β	*t*	*p*
Constant (DQI-I Score)	38.082	[45.467, 70.227]	–	5.215	< 0.001
Sex / Gender (male / female)	−1.902	[−9.052, 2.683]	−0.096	−0.780	0.439
Age (years)	0.218	[0.043, 0.271]	0.227	1.779	0.080
FEV_1_%	0.114	[−13.107, 1.855]	0.293	2.330	0.023
Modulator use (yes / no)	−2.716	[−12.496, 1.352]	−0.110	−0.901	0.371

^*^p < 0.05. **p < 0.01. *n* = 67

R^2^adj = 0.075 (*n* = 67, *p* = 0.065)

Abbreviations: DQI-I, Diet Quality Index– International; FEV_1_%, predicted percentage forced expiratory volume; CI, confidence interval of *B*

#### PROM associations and predictors of HEI-2020 and DQI-I diet quality scores

Pearson’s correlations revealed significant and moderate positive associations between HEI-2020 and physical functioning (CFQ-R) (*R* = 0.323,* p* = 0.005), emotional functioning (CFQ-R) (*R* = 0.308, *p* = 0.007), health perceptions (CFQ-R) (*R* = 0.336, *p* = 0.003), EuroQol Visual Analogue Scale score (*R* = 0.362, *p* = 0.002) and EQ-5D-5L Irish index value (*R* = 0.306, *p* = 0.007). Within the final presented linear regression model, the variables significantly predicting HEI-2020 were FEV_1_% and digestive symptoms (CFQ-R) (Table 5).

**Table 5:** Regression 3: Pearson’s correlations amongst the dependent (HEI-2020) and independent variables and coefficients for predicted HEI-2020 scores in adults living with CF

Regression 3 (includes PROMs)	1	2	3	4	5
1. HEI-2020 Score (0–100)	–	0.332**	0.221*	0.161	0.341**
2. FEV_1_%		**–**	−0.122	0.271	0.491**
3. Digestive Symptoms (CFQ-R)			**–**	0.476**	0.229*
4. Vitality (CFQ-R)				**–**	0.570**
5. Physical Functioning (CFQ-R)					**–**

Variable	*B*	95% CI	β	*t*	*p*
Constant (HEI-2020 Score)	31.546	[16.378, 46.715]	**–**	4.157	< 0.001
FEV_1_%	0.153	[0.018, 0.288]	0.307	2.270	0.027
Digestive Symptoms (CFQ-R)	0.182	[0.019, 0.345]	0.299	2.228	0.030
Vitality (CFQ-R)	−0.126	[−0.320, 0.069]	−0.198	−1.293	0.201
Physical Functioning (CFQ-R)	0.118	[−0.35, 0.270]	0.234	1.544	0.128

R^2^adj = 0.166 (*n* = 67, *p* = 0.004)

Abbreviations: PROMs, patient reported outcome measures; HEI-2020, Health Eating Index– 2020; FEV_1_%, predicted percentage forced expiratory volume; CFQ-R, Cystic Fibrosis Questionnaire– Revised; CI, confidence interval of *B*

Pearson’s correlations revealed a significant and moderate positive association between DQI-I and emotional functioning (CFQ-R) (*R* = 0.303, *p* = 0.008). After performing multiple linear regressions with DQI-I as the dependent variable, no model or PROM variable were identified as predictors for DQI-I scores (data not shown).

## Discussion

This study examines HRQoL and predictors of diet quality in PwCF at a time when CFTR modulators are changing the phenotypic presentation of CF in the acute and outpatient settings. To the best of the authors’ knowledge, this study presents a novel contribution to the field, evaluating real life influences on diet quality amongst adult PwCF, with findings essential for shaping guideline changes and guiding effective interventions to mitigate the burden of diet-related chronic disease in PwCF. Results indicate in the modulator era of CF, HRQoL symptoms related to suboptimal vitality, anxiety / depression, and gut symptoms remain prevalent among adult PwCF, and both FEV_1_% and digestive symptoms (CFQ-R) presented as predictors of diet quality (HEI-2020).

### HRQoL and symptom severity in adult PwCF

Irish adult PwCF report positive HRQoL across the majority of HRQoL domains assessed. In line with previous research [47–50], the CFQ-R indicates that eating problems rank as the highest scoring domain, while vitality ranks the lowest in PwCF both above and below mean HEI-2020 and DQI-I scores. However, differing from previous research [47–50], the median CFQ-R weight domain also scores highest. This result likely reflects changing priorities in a healthy CF population, whereby gaining weight is no longer as challenging due to advancements in modulator therapies and clinical practices [2, 4–6]. Also notable, the median scores for vitality (both above and below mean HEI-2020 and DQI-I scores) are higher than overall scores for cohorts reported in earlier studies [47–50]. This diminished sense of wellbeing is echoed in EQ-5D-5L scores where high incidences of anxiety / depression and pain / discomfort symptoms were reported. Anxiety / depression and pain / discomfort are deeply intertwined, each capable of exacerbating the other and undermining feelings of vitality through increased fatigue and lack of motivation and disrupted sleep and emotional wellbeing [51]. Compared to findings on EQ-5D-5L dimensions in a representative sample (*N* = 1131) of the Irish general population, there are similarities in that anxiety / depression and pain / discomfort are the most commonly reported symptoms. However, adult PwCF have almost twice the prevalence of slight to extreme symptoms of anxiety / depression, while also reporting lower prevalences of slight to extreme symptoms of pain / discomfort to that of the Irish population sampled [52]. These findings are relevant as while CFTR modulators have revolutionised CF treatment, leading to substantial improvements in clinical outcomes [2, 3], the psychosocial and economic burdens of CF nonetheless still remain and PwCF continue to face difficulties in relation to disease identity and societal roles, which are underexplored [53]. Further research is necessary to address knowledge gaps and ensure comprehensive care is inclusive of psychosocial and long-term health considerations.

Gut symptoms are highly prevalent in the current study with the majority of participants reporting symptoms of stomach bloating, discomfort, fullness and feeling visibly larger. Moderate to very severe symptoms are common within the cohort with PAC-SYM ‘bloating in stomach’ being the most prevalent. Moshiree et al*.* [54] similarly reported high prevalences of moderate to very severe gut symptoms in PwCF. However, proportions of moderate to very severe PAGI-SYM bloating, stomach fullness and stomach or belly visibly larger symptoms were higher than prevalences reported in the current study.

While adult PwCF report less gut symptoms compared to those in the general population with irritable bowel syndrome (PAGI-SYM score: 1.3 ± 0.9) and functional diarrhoea (PAGI-SYM score: 0.87 ± 0.6) [55], they experience similar rates of abdominal bloating and have unique symptom profiles that warrant further investigation. In addition, within the general population cohort an overlap was observed between gut and psychosomatic symptoms [55]. A bidirectional relationship exists between the gut and central nervous system with gut microbiota playing a significant role in predictions of mental health and wellbeing within dietary interventions [56]. With poor diet quality reported in Irish adult PwCF, future research should endeavour to assess the effect of an appropriate dietary intervention on HRQoL domains inclusive of feelings of vitality, gut, and anxiety / depression symptoms in adult PwCF.

### Interrelationships between clinical variables, PROMs and diet quality

When evaluating PROM predictors of diet quality scores (HEI-2020 / DQI-I), while the digestive symptoms (CFQ-R) domain is identified as an independently positive significant predictor of HEI-2020 scores alongside FEV_1_%, no PROMs as assessed were significant predictors for DQI-I scores. General population studies have explored the relationships between diet quality, and digestive symptoms and gastrointestinal disorders which highlighted that more adverse digestive symptoms are associated with poorer diet quality, higher intakes of ultra processed foods, and adherence to a more Westernised dietary pattern [57–60]. While recent research reported significant improvements in abdominal symptoms following 1-year of elexacaftor-tezacaftor-ivacaftor treatment as measured by a novel and CF-validated abdominal symptoms questionnaire, the CFAbd-Score [61], dietary constituents were not accounted for, which may explain a potential mechanism of improvement.

FEV_1_% is identified as an independently positive significant predictor of HEI-2020, and while a combination of the variables within the model do not provide a significant explanation for variations in DQI-I scores, FEV_1_% alone is associated with changes in the DQI-I score. While a predictive relationship exists, it is possible that this is indirect, resulting from changing dietary priorities as CF health concerns improve, whereby the primary focus may have shifted from survival to prevention of chronic disease progression, reflected in recent literature [62]. Likewise, the Mediterranean Diet Score has been independently associated with FEV_1_% (*p* = 0.002) in adults with chronic obstructive pulmonary disease [63]. For both populations, it may be that with improved lung function individuals are likely feeling better and have more energy to make nutritious meals and focus on achieving an overall healthier diet.

A cross-sectional study investigating associations of adherence to the Mediterranean diet with gut microbiota characteristics and gastrointestinal symptomatology in adults in the general population (*n* = 116) discovered that participants with a high adherence had more favourable gut microbiota including lower counts of *E. Coli* (*p* = 0.022), a higher Bifidobacteria*: E*. *coli* ratio (*p* = 0.025), higher *C. albicans* levels (*p* = 0.03) and prevalences (*p* = 0.05), and higher defaecation frequency (low adherence: 7.0 (3.3) / week; high adherence: 9.0 (5.0) / week, *p* = 0.028) compared to individuals with low adherence, which was interpreted as a favourable functional outcome. While participants with high adherence reported a more pronounced gastrointestinal symptomatology compared to individuals with a low adherence, these symptoms were reported as mild and transient, often associated with increases in fibre intake which provides an overall improvement to the gut microbiome [64]. Given the predictive nature of digestive symptoms and the severity of symptoms reported in adult PwCF, it is crucial that dietary approaches be adopted to address issues related to gut symptoms and the gut microbiota. A recent review highlighted that to date, no research exists exploring the relationship between gut symptoms and the CF gut microbiome and supported an exploration of the impact of diet on the gut microbiota in PwCF [65]. With evidence outside of CF supporting an association between improvements to the gut microbiome and Mediterranean diet adherence, similar dietary approaches could be trialled in PwCF, following the implementation of dietary intervention studies that assess the dietary patterns effectiveness in a CF context.

With only a moderate correlation observed between HEI-2020 and DQI-I and a lack of homogeneity between the diet quality scores regarding their predictors, the indices do not appear translatable in a CF context. While both diet quality indices have previously been associated with metabolic risk factors [66, 67] increasingly relevant in CF, HEI-2020 is specifically designed to follow the most up to date general population nutrition guidelines (USA) in producing a score [36], and the latest CF nutrition guidelines [1], recommend most PwCF should follow nutrition guidelines similar to the general population. Furthermore, HEI-2020 and has been previously used in and adult CF settings [25, 38]. Thus, HEI-2020 may be a more appropriate tool to assess diet quality in PwCF within a research context. The application of each diet quality score is labour intensive and potentially time consuming making widespread implementation of either diet quality score unlikely to be feasible within clinical settings where time constraints may exist. Comparisons of dietary intakes with national and regional nutrition guidelines may be best practice to evaluate diet quality in clinical settings moving forward.

### Strengths and limitations

The cohort in this study is broadly representative of the future CF population of Western countries [44, 68]. The range of PROMs evaluated are extensive, capturing a wide variety of symptoms comparable within and outside the CF context. However, in the modulator treated CF population, there are concerns related to the relevance of certain PROMs including the CFQ-R and the potential for ceiling effects [22], with questions more appropriate for the traditionally malnourished CF population. Furthermore, while PAGI-SYM and PAC-SYM have been widely used in CF research, in recent research the CFAbd-Score has taken place of the questionnaires. Nonetheless, the PAGI-SYM and PAC-SYM provide additional value in that scores are comparable to non-CF populations and allow for direct comparison to pre-modulator era CF data. Although some participants included in the study were not receiving CFTR modulators, exploratory analyses (Supplementary Material 1 and 2) [15] indicated that their inclusion did not skew overall findings. Nonetheless, the imbalance in sample size between modulator users and non-users warrants acknowledgment. Future research may benefit from stratified recruitment or dedicated subgroup analyses to more rigorously investigate potential differences by modulator status. Although the study did not include a comparator group, the authors sought to contextualise findings by making comparisons with pre- and post-modulator era CF populations [47–50, 54, 61], the general population [52, 57–60, 64], and where relevant, patients living with chronic obstructive pulmonary disease [63], irritable bowel syndrome, and functional diarrhoea [55]. While the sample size is modest, statistical procedures were carefully applied to reduce the risk of overfitting. Final regression models contained a limited number of variables (*n* ≤ 5) in line with sample size recommendations. Given the modest sample size, future research should aim to replicate this study in a larger cohort with an appropriate comparator group to strengthen the findings and yield a more robust effect size.

## Conclusion

This cross-sectional study highlights that in the modulator era, HRQoL symptoms related to suboptimal vitality, anxiety / depression, and gut symptoms remain prevalent among adult PwCF. Diet quality was positively associated with FEV_1_% and digestive symptoms, supporting the need for dietary interventions focused on long-term health, encompassing dietary patterns associated with improvements in the gut microbiome and gut symptoms. With a bidirectional relationship between the gut and central nervous system existing, this may contribute to improvements in psychosomatic symptoms in PwCF. Nonetheless, additional strides should be made to provide comprehensive care inclusive of both psychosocial and long-term health considerations. With notable time constraints existing with the application of diet quality indices, tools as such are likely only applicable within research settings. Future research should endeavour to validate findings in a representative sample of adult PwCF. Patient-informed dietary intervention studies are necessary to inform future nutrition guidelines and clinical practices on the optimal dietary pattern for PwCF incorporating concerns related to diet-related chronic disease and gut symptoms.

## Electronic supplementary material

Below is the link to the electronic supplementary material.

Supplementary Material 1

Supplementary Material 2

Supplementary Material 3

## Data Availability

The data that support the findings of this study are available from the corresponding author upon reasonable request.

## Abbreviations

CF: Cystic fibrosis
CFTR: Cystic fibrosis transmembrane conductance regulator
PwCF: People living with cystic fibrosis
BMI: Body mass index
FEV_1_%: Predicted percentage forced expiratory volume
HRQoL: Health-related quality of life
PROMs: Patient-reported outcome measures
CFQ-R: Cystic fibrosis questionnaire-revised
EQ-5D-5L: EuroQol 5-dimension 5-level questionnaire
PAGI-SYM: Patient assessment of gastrointestinal symptoms
PAC-SYM: Patient assessment of constipation symptoms
EDC: Electronic data capture system
HEI-2020: Healthy eating index-2020
DQI-I: Diet quality index-international
