# Supplementary material for: Interrelationships between diet quality and health-related quality of life in Irish adults living with cystic fibrosis
Source: Eur J Nutr. 2025 Jul 24;64(6):248. doi: 10.1007/s00394-025-03766-y (PMC12289792; doi:10.1007/s00394-025-03766-y)
Supplement: Supplementary file 1 — Supplementary Material 1 [file 394_2025_3766_MOESM1_ESM.docx]

1. PROM subgroup analysis

*1.1 CFQ-R*

Male participants had significantly higher emotional functioning (78.7 ± 17.2, *p* = 0.038) and eating problems (96.9 ± 8.6, *p* = 0.027) domain scores compared to female participants (emotional functioning; 70.6 ± 19.6; eating problems: 90.8 ± 14.7) (Table S1). Participants classified as overweight or obese had significantly higher domain scores for eating problems (98.0 ± 5.3, *p* = 0.021), and weight (97.6 ± 12.6, *p* = 0.009) compared to participants classified as underweight or normal (eating problems: 90.6 ± 15.0; weight: 84.4 ± 28.1) (See Table S2). Participants classified as PI had significantly lower domain scores for physical functioning (78.2 ± 25.5, *p* = 0.028), treatment burden (68.9 ± 120.6, *p* = 0.010), health perceptions (72.3 ± 24.7, *p* = 0.019), social / school functioning (71.2 ± 19.0, *p* = 0.033), body image (73.1 ± 24.4, *p* = 0.041), and role functioning (86.5 ± 17.6, *p* = 0.028) compared to PS participants (physical functioning: 89.7 ± 18.2; treatment burden: 83.0 ± 20.1; health perceptions: 86.3 ± 13.9; social / school functioning: 82.0 ± 18.0; body image: 85.0 ± 20.8; role functioning: 93.6 ± 12.0) (Table S3). Participants above the mean FEV_1_% scored significantly higher in domains for physical functioning (86.7 ± 19.2, *p* = 0.009) and health perceptions (81.7 ± 16.4, *p* = 0.035) compared to participants below the mean FEV_1_% (physical functioning: 72.8 ± 28.5; health perceptions: 67.0 ± 28.7) (See Table S4). Participants taking modulators scored significantly lower in the domain for social / school functioning (71.0 ± 19.4, *p* = 0.011) compared to participants not taking modulators (84.4 ± 14.9) (See Table S5). Participants on a part-time income (*n* = 15) had significantly lower domain scores for vitality (51.8 ± 19.7, sig.^adj.^ = 0.023), emotional functioning (60.5 ± 23.7, sig.^adj.^ = 0.009), treatment burden (60.3 ± 22.5, sig.^adj.^ = 0.030), body image (56.3 ± 28.7, sig.^adj.^ = 0.002), and role functioning (83.3 ± 16.3, sig.^adj.^ = 0.018) compared to participants with a full income (*n* = 33) (vitality: 70.5 ± 15.3; emotional functioning: 80.6 ± 12.3; treatment burden: 78.8 ± 18.7; body image: 84.5 ± 20.0; and role functioning: 94.2 ± 9.0) (Table S6).

*1.2 EQ-5D-5L*

Participants above the mean FEV_1_% reported a better EQ-5D-5L mobility dimension score (1.1 ± 0.4, *p* = 0.045) compared to participants below the mean FEV_1_% (1.3 ± 0.7) (See Table S4). Participants taking modulators had significantly lower EQ-5D-5L pain / discomfort dimension scores (1.5 ± 074, *p* = 0.022) compared to participants not taking modulators (1.1 ± 0.3) (Table S5). Participants with a part-time income reported a significantly worse scores for the dimension of anxiety / depression (2.0 ± 0.9, sig.^adj.^ = 0.040) compared to participants on a full income (1.4 ± 0.5) (Table S6).

*1.3 PAC-SYM and PAGI-SYM*

Male participants had significantly better aggregated PAC-SYM scores (0.3 ± 0.3, *p* = 0.030) compared to female participants (0.6 ± 0.6) (Table S1). Participants with PS reported significantly better final PAGI-SYM score (0.3 ± 0.5, *p* = 0.015) compared to PI participants (0.6 ± 0.6) (Table S3). In relation to PAGI-SYM subscale scores, female participants reported significantly more symptoms of nausea / vomiting (0.3 ± 0.4, *p* = 0.005), bloating (1.5 ± 1.4, *p* = 0.035), and lower-abdominal pain (0.6 ± 0.9, *p* = 0.021) compared to male participants (nausea / vomiting: 0.1 ± 0.3; bloating: 0.8 ± 0.9; lower-abdominal pain: 0.1 ± 0.3) (Table S1). PI participants reported significantly more symptoms of heartburn / regurgitation (0.4 ± 0.6, *p* = 0.005) and upper-abdominal pain (0.4 ± 0.8, *p* = 0.019) compared to PS participants (heartburn / regurgitation: 0.1 ± 0.2; upper-abdominal pain: 0.0 ± 0.0) (Table S3). Participants on a full income scored significantly better in the PAGI-SYM subscales of heartburn / regurgitation (0.1 ± 0.2, sig.^adj.^ = 0.001) and bloating (0.8 ± 0.8, sig.^adj.^ = 0.015) compared to participants on a part-time income (heartburn / regurgitation:0.7 ± 0.9; bloating: 1.8 ± 1.1) (Table S 8).

Table S1. PROMs (CFQ-R, EQ-5D-5L, PAC-SYM and PAGI-SYM) in adults living with CF for total cohort, males and females.

| **PROM** | **All (*N* = 73)** | **Male (*n* = 32)** | **Female (*n* = 41)** | ***p*-values** |
| --- | --- | --- | --- | --- |
| **CFQ-R Domain Scores (0-100)** |  |  |  |  |
| *Physical Functioning* | 91.7 (29.2) | 89.6 (29.2) | 95.8 (31.3) | 0.581 |
| *Vitality* | 66.7 (25.0) | 66.7 (16.7) | 66.7 (25.0) | 0.131 |
| *Emotional Functioning* | 73.3 (20.0) | 80.0 (18.3) | 73.3 (26.7) | 0.038 |
| *Eating Problems* | 100.0 (11.1) | 100.0 (0.0) | 100.0 (11.1) | 0.027 |
| *Treatment Burden* | 77.8 (33.3) | 83.3 (33.3) | 66.7 (27.8) | 0.249 |
| *Health Perceptions* | 77.8 (22.2) | 77.8 (22.2) | 77.8 (33.3) | 0.314 |
| *Social/School Functioning* | 72.2 (33.3) | 80.6 (26.4) | 72.2 (38.9) | 0.553 |
| *Body Image* | 77.8 (44.5) | 88.9 (33.3) | 77.8 (44.5) | 0.486 |
| *Role Functioning* | 91.7 (16.7) | 100.0 (16.7) | 91.7 (16.7) | 0.185 |
| *Weight* | 100.0 (0.0) | 100.0 (0.0) | 100.0 (0.0) | 0.544 |
| *Respiratory Symptoms* | 94.4 (16.7) | 94.4 (16.7) | 88.9 (11.1) | 0.209 |
| *Digestive Symptoms* | 88.9 (33.3) | 88.9 (30.6) | 77.8 (33.3) | 0.991 |
|  | ***n* = 72** | ***n* = 32** | ***n* = 40** |  |
| **EQ-5D-5L Total Scores** |  |  |  | 0.643 |
| *11111 n(%)* | 34 (47.2) | 16 (50.0) | 18 (43.9) |  |
| *any other health state n(%)* | 38 (52.8) | 16 (50.0) | 23 (56.1) |  |
| *EQ Visual Analogue Scale score (0-100)* | 82.0 (15.0) | 80.5 (15.0) | 85.0 (10.0) | 0.636 |
| ***Having any problem in five dimensions (score* ≥*2) n(%)*** | |  |  |  |
| *Mobility* | 10 (13.9) | 3 (9.4) | 7 (17.5) | 0.777 |
| *Self-care* | 2 (2.8) | 1 (3.1) | 1 (2.5) | 1.000 |
| *Usual Activities***^~^** | 9 (12.5) | 5 (15.6) | 4 (10.0) | 0.754 |
| *Pain / Discomfort* | 22 (30.6) | 7 (21.9) | 15 (37.5) | 0.605 |
| *Anxiety / Depression Severity* | 31 (43.1) | 12 (37.5) | 19 (47.5) | 0.097 |
| ***Dimension scores (1-5)*** |  |  |  |  |
| *Mobility (Walking)* | 1.0 (0.0) | 1.0 (0.0) | 1.0 (0.0) | 0.326 |
| *Self-care (washing & dressing)* | 1.0 (0.0) | 1.0 (0.0) | 1.0 (0.0) | 0.873 |
| *Usual Activities (e.g., work, study, housework, family or leisure activities)* | 1.0 (0.0) | 1.0 (0.0) | 1.0 (0.0) | 0.489 |
| *Pain / Discomfort* | 1.0 (1.0) | 1.0 (0.0) | 1.0 (1.0) | 0.241 |
| *Anxiety / Depression Severity* | 1.0 (1.0) | 1.0 (1.0) | 1.0 (1.0) | 0.188 |
|  |  | ***n* = 31** | ***n* = 41** |  |
| **PAC-SYM Score (0-4)** | 0.3 (0.7) | 0.2 (0.4) | 0.5 (0.7) | 0.030 |
| ***Subscales*** |  |  |  |  |
| *Abdominal Symptoms* | 0.5 (1.0) | 0.5 (0.8) | 0.8 (1.4) | 0.085 |
| *Rectal Symptoms* | 0.0 (0.3) | 0.0 (0.3) | 0.0 (0.3) | 0.575 |
| *Stool Symptoms* | 0.2 (0.6) | 0.1 (0.4) | 0.4 (0.8) | 0.138 |
| ***Constipation symptom details*** |  |  |  |  |
| *discomfort in stomach* | 1.0 (1.0) | 0.0 (1.0) | 1.0 (2.0) | 0.125 |
| *pain in your stomach* | 0.0 (1.0) | 0.0 (1.0) | 0.0 (1.0) | 0.096 |
| *bloating in your stomach* | 1.0 (2.0) | 1.0 (2.0) | 2.0 (2.0) | 0.110 |
| *stomach cramps* | 0.0 (1.0) | 0.0 (0.0) | 0.0 (1.0) | 0.022 |
| *painful bowel movements* | 0.0 (0.0) | 0.0 (0.0) | 0.0 (0.5) | 0.599 |
| *rectal burning during / after a bowel movement* | 0.0 (0.0) | 0.0 (0.0) | 0.0 (0.0) | 0.104 |
| *rectal bleeding or tearing during / after a bowel movement* | 0.0 (0.0) | 0.0 (0.0) | 0.0 (0.0) | 0.806 |
| *incomplete bowel movement, felt like you didn't finish* | 0.0 (1.0) | 0.0 (1.0) | 0.0 (1.0) | 0.648 |
| *bowel movements were too hard* | 0.0 (0.0) | 0.0 (0.0) | 0.0 (0.5) | 0.386 |
| *bowel movements were too small* | 0.0 (1.0) | 0.0 (0.3) | 0.0 (1.0) | 0.155 |
| *straining or squeezing to try to pass bowel movements* | 0.0 (1.0) | 0.0 (0.0) | 0.0 (1.0) | 0.215 |
| *feeling like you had to pass a bowel movement but you could not ('false alarm')* | 0.0 (0.0) | 0.0 (0.0) | 0.0 (0.8) | 0.206 |
|  | ***n* = 70** | ***n* = 30** | ***n* = 40** |  |
| **PAGI-SYM Score (0-5)** | 0.3 (0.6) | 0.3 (0.6) | 0.4 (1.1) | 0.065 |
| ***Subscales*** |  |  |  |  |
| *Heartburn / Regurgitation* | 0.1 (0.3) | 0.0 (0.3) | 0.1 (0.3) | 0.389 |
| *Nausea / Vomiting* | 0.0 (0.3) | 0.0 (0.0) | 0.0 (0.3) | 0.005 |
| *Fullness / Satiety* | 0.5 (1.0) | 0.3 (0.8) | 0.5 (1.2) | 0.183 |
| *Bloating* | 1.0 (2.0) | 0.8 (1.6) | 1.0 (2.0) | 0.035 |
| *Upper-Abdominal Pain* | 0.0 (0.0) | 0.0 (0.1) | 0.0 (0.0) | 0.686 |
| *Lower-Abdominal Pain* | 0.0 (0.5) | 0.0 (0.0) | 0.0 (1.0) | 0.021 |
| ***Gastrointestinal symptom details*** |  |  |  |  |
| *Nausea* | 0.0 (1.0) | 0.0 (0.0) | 0.0 (1.0) | 0.006 |
| *Retching* | 0.0 (0.0) | 0.0 (0.0) | 0.0 (0.0) | 0.642 |
| *Vomiting* | 0.0 (0.0) | 0.0 (0.0) | 0.0 (0.0) | 0.735 |
| *Stomach fullness* | 1.0 (2.0) | 1.0 (1.5) | 1.0 (2.0) | 0.072 |
| *Not able to finish a normal sized meal* | 0.0 (1.0) | 0.0 (0.3) | 0.0 (1.0) | 0.626 |
| *Feeling excessively full after meals* | 0.0 (1.0) | 0.0 (1.0) | 0.0 (2.0) | 0.989 |
| *Loss of appetite* | 0.0 (0.3) | 0.0 (0.0) | 0.0 (1.0) | 0.224 |
| *Bloating* | 1.0 (2.0) | 1.0 (2.0) | 1.0 (2.0) | 0.094 |
| *Stomach or belly visibly larger* | 1.0 (2.0) | 0.0 (2.0) | 1.0 (2.8) | 0.041 |
| *Upper abdominal pain* | 0.0 (0.0) | 0.0 (0.0) | 0.0 (0.0) | 0.984 |
| *Upper abdominal discomfort* | 0.0 (0.0) | 0.0 (0.0) | 0.0 (0.0) | 0.372 |
| *Lower abdominal pain* | 0.0 (0.0) | 0.0 (0.0) | 0.0 (0.0) | 0.143 |
| *Lower abdominal discomfort* | 0.0 (1.0) | 0.0 (0.0) | 0.0 (1.0) | 0.011 |
| *Heartburn during the day* | 0.0 (0.0) | 0.0 (0.0) | 0.0 (1.0) | 0.232 |
| *Heartburn when lying down* | 0.0 (0.0) | 0.0 (0.0) | 0.0 (1.0) | 0.149 |
| *Discomfort inside your chest during the day* | 0.0 (0.0) | 0.0 (0.0) | 0.0 (0.0) | 0.674 |
| *Discomfort inside your chest at night* | 0.0 (0.0) | 0.0 (0.0) | 0.0 (0.0) | 0.445 |
| *Regurgitation or reflux during the day* | 0.0 (0.0) | 0.0 (0.3) | 0.0 (0.0) | 0.994 |
| *Regurgitation or reflux when lying down* | 0.0 (0.0) | 0.0 (0.3) | 0.0 (0.0) | 0.298 |
| *Bitter, acid or sour taste in your mouth* | 0.0 (0.0) | 0.0 (0.0) | 0.0 (0.0) | 0.660 |
| Values are represented as median (interquartile range) or *n* (%).  *P*-values were derived with chi-square tests, Mann Whitney U and Kruskal-Wallis tests (non-parametric).  Significance derived from a *p*-value of <0.05.  **^~^**Work, study, housework, family or leisure activities.  Abbreviations: PROM Patient-reported outcome measure, CFQ-R Cystic Fibrosis Questionnaire – Revised, EQ-5D-5L EuroQol 5-dimension 5-level questionnaire, EQ EuroQol, PAC-SYM Patient Assessment of Constipation Symptoms questionnaire, PAGI-SYM Patient Assessment of Upper Gastrointestinal Symptoms questionnaire. | | | | |

Table S2. PROMs (CFQ-R, EQ-5D-5L, PAC-SYM and PAGI-SYM) in adults living with CF by BMI.

| **PROM** | **≤ 24.9 kg/m^2^**  **(*n* = 45)** | **≥ 25 kg/m^2^***  **(*n* = 28)** | ***p*-values** |  |
| --- | --- | --- | --- | --- |
| **CFQ-R Domain Scores (0-100)** |  |  |  |  |
| *Physical Functioning* | 95.8 (29.2) | 87.5 (39.6) | 0.122 |  |
| *Vitality* | 66.7 (25.0) | 62.5 (31.2) | 0.743 |  |
| *Emotional Functioning* | 80.0 (20.0) | 73.3 (25.0) | 0.968 |  |
| *Eating Problems* | 100.0 (11.1) | 100.0 (0.0) | 0.021 |  |
| *Treatment Burden* | 77.8 (33.3) | 77.8 (33.3) | 0.734 |  |
| *Health Perceptions* | 77.8 (22.2) | 77.8 (30.6) | 0.296 |  |
| *Social/School Functioning* | 77.8 (30.6) | 66.7 (33.3) | 0.193 |  |
| *Body Image* | 77.8 (44.5) | 83.3 (33.3) | 0.358 |  |
| *Role Functioning* | 91.7 (16.7) | 91.7 (22.9) | 0.130 |  |
| *Weight* | 100.0 (33.3) | 100.0 (0.0) | 0.009 |  |
| *Respiratory Symptoms* | 88.9 (13.9) | 94.4 (20.8) | 0.401 |  |
| *Digestive Symptoms* | 88.9 (33.3) | 77.8 (22.2) | 0.414 |  |
|  | **n = 44** | **n = 28** |  |  |
| **EQ-5D-5L Total Scores** |  |  | 0.632 |  |
| *11111 n(%)* | 22 (50.0) | 12 (42.9) |  |  |
| *any other health state n(%)* | 22 (50.0) | 16 (57.1) |  |  |
| *EQ Visual Analogue Scale score (0-100)* | 85.0 (10.0) | 80.0 (15.0) | 0.326 |  |
| ***Having any problem in five dimensions (score* ≥*2) n(%)*** | | | | |
| *Mobility* | 6 (13.6) | 4 (14.3) | 1.000 |  |
| *Self-care* | 0 (0.0) | 2 (7.1) | 0.148 |  |
| *Usual Activities***^~^** | 4 (9.1) | 5 (17.9) | 0.297 |  |
| *Pain / Discomfort* | 13 (29.5) | 9 (32.1) | 1.000 |  |
| *Anxiety / Depression Severity* | 17 (31.5) | 14 (50.0) | 0.464 |  |
| ***Dimension scores (1-5)*** |  |  |  |  |
| *Mobility (Walking)* | 1.0 (0.0) | 1.0 (0.0) | 0.939 |  |
| *Self-care (washing & dressing)* | 1.0 (0.0) | 1.0 (0.0) | 0.074 |  |
| *Usual Activities***^~^** | 1.0 (0.0) | 1.0 (0.0) | 0.277 |  |
| *Pain / Discomfort* | 1.0 (1.0) | 1.0 (1.0) | 0.694 |  |
| *Anxiety / Depression Severity* | 1.0 (1.0) | 1.5 (1.0) | 0.462 |  |
|  | ***n* = 45** | ***n* = 27** |  |  |
| **PAC-SYM Score (0-4)** | 0.3 (0.7) | 0.4 (0.6) | 0.971 |  |
| ***Subscales*** |  |  |  |  |
| *Abdominal Symptoms* | 0.5 (1.3) | 0.5 (1.0) | 0.663 |  |
| *Rectal Symptoms* | 0.0 (0.3) | 0.0 (0.3) | 0.931 |  |
| *Stool Symptoms* | 0.2 (0.6) | 0.0 (0.7) | 0.499 |  |
| ***Constipation symptom details*** |  |  |  |  |
| *discomfort in stomach* | 1.0 (1.0) | 1.0 (2.0) | 0.875 |  |
| *pain in your stomach* | 0.0 (1.0) | 0.0 (1.0) | 0.850 |  |
| *bloating in your stomach* | 1.0 (2.0) | 1.0 (1.0) | 0.417 |  |
| *stomach cramps* | 0.0 (1.0) | 0.0 (1.0) | 0.595 |  |
| *painful bowel movements* | 0.0 (0.0) | 0.0 (0.0) | 0.981 |  |
| *rectal burning during / after a bowel movement* | 0.0 (0.0) | 0.0 (0.0) | 0.178 |  |
| *rectal bleeding or tearing during / after a bowel movement* | 0.0 (0.0) | 0.0 (0.0) | 0.594 |  |
| *incomplete bowel movement, felt like you didn't finish* | 0.0 (1.0) | 0.0 (1.0) | 0.913 |  |
| *bowel movements were too hard* | 0.0 (0.0) | 0.0 (0.0) | 0.700 |  |
| *bowel movements were too small* | 0.0 (1.0) | 0.0 (0.0) | 0.032 |  |
| *straining or squeezing to try to pass bowel movements* | 0.0 (1.0) | 0.0 (1.0) | 0.698 |  |
| *feeling like you had to pass a bowel movement but you could not ('false alarm')* | 0.0 (0.0) | 0.0 (1.0) | 0.327 |  |
|  | ***n* = 44** | ***n* = 26** |  |  |
| **PAGI-SYM Score (0-5)** | 0.3 (0.6) | 0.5 (0.6) | 0.588 |  |
| ***Subscales*** |  |  |  |  |
| *Heartburn / Regurgitation* | 0.0 (0.3) | 0.1 (0.6) | 0.223 |  |
| *Nausea / Vomiting* | 0.0 (0.3) | 0.0 (0.1) | 0.501 |  |
| *Fullness / Satiety* | 0.3 (1.0) | 0.5 (1.0) | 0.995 |  |
| *Bloating* | 1.0 (2.0) | 1.0 (2.0) | 0.937 |  |
| *Upper-Abdominal Pain* | 0.0 (0.0) | 0.0 (0.5) | 0.343 |  |
| *Lower-Abdominal Pain* | 0.0 (0.5) | 0.0 (0.5) | 0.622 |  |
| ***Gastrointestinal symptom details*** |  |  |  |  |
| *Nausea* | 0.0 (1.0) | 0.0 (0.3) | 0.497 |  |
| *Retching* | 0.0 (0.0) | 0.0 (0.0) | 0.499 |  |
| *Vomiting* | 0.0 (0.0) | 0.0 (0.0) | 0.283 |  |
| *Stomach fullness* | 1.0 (2.0) | 1.0 (2.0) | 0.927 |  |
| *Not able to finish a normal sized meal* | 0.0 (1.0) | 0.0 (0.3) | 0.837 |  |
| *Feeling excessively full after meals* | 0.0 (1.0) | 0.0 (1.5) | 0.498 |  |
| *Loss of appetite* | 0.0 (1.0) | 0.0 (0.0) | 0.418 |  |
| *Bloating* | 1.0 (2.0) | 1.0 (2.0) | 0.934 |  |
| *Stomach or belly visibly larger* | 1.0 (2.0) | 1.0 (2.0) | 0.456 |  |
| *Upper abdominal pain* | 0.0 (0.0) | 0.0 (0.0) | 0.810 |  |
| *Upper abdominal discomfort* | 0.0 (0.0) | 0.0 (1.0) | 0.312 |  |
| *Lower abdominal pain* | 0.0 (0.0) | 0.0 (0.0) | 0.718 |  |
| *Lower abdominal discomfort* | 0.0 (1.0) | 0.0 (1.0) | 0.812 |  |
| *Heartburn during the day* | 0.0 (0.0) | 0.0 (0.3) | 0.882 |  |
| *Heartburn when lying down* | 0.0 (0.0) | 0.0 (1.0) | 0.410 |  |
| *Discomfort inside your chest during the day* | 0.0 (0.0) | 0.0 (1.0) | 0.009 |  |
| *Discomfort inside your chest at night* | 0.0 (0.0) | 0.0 (0.3) | 0.006 |  |
| *Regurgitation or reflux during the day* | 0.0 (0.0) | 0.0 (1.0) | 0.437 |  |
| *Regurgitation or reflux when lying down* | 0.0 (0.0) | 0.0 (1.0) | 0.082 |  |
| *Bitter, acid or sour taste in your mouth* | 0.0 (0.0) | 0.0 (0.3) | 0.395 |  |
| Values are represented as median (interquartile range) or *n* (%).  *P*-values were derived with chi-square tests, Mann Whitney U and Kruskal-Wallis tests (non-parametric).  Significance derived from a *p*-value of <0.05.  *World Health Organisation cut-off values for individuals overweight/obese (52).  **^~^**Work, study, housework, family or leisure activities.  Abbreviations: PROM Patient-reported outcome measure, CFQ-R Cystic Fibrosis Questionnaire – Revised, EQ-5D-5L EuroQol 5-dimension 5-level questionnaire, EQ EuroQol, PAC-SYM Patient Assessment of Constipation Symptoms questionnaire, PAGI-SYM Patient Assessment of Upper Gastrointestinal Symptoms questionnaire. | | | |  |

Table S3. PROMs (CFQ-R, EQ-5D-5L, PAC-SYM and PAGI-SYM) in adults living with CF by pancreatic status.

| **PROM** | **PI (*n* = 55)** | **PS (*n* = 17)** | ***p*-values** |  |
| --- | --- | --- | --- | --- |
| **CFQ-R Domain Scores (0-100)** |  |  |  |  |
| *Physical Functioning* | 87.5 (33.3) | 100.0 (14.6) | 0.028 |  |
| *Vitality* | 66.7 (25.0) | 75.0 (20.8) | 0.110 |  |
| *Emotional Functioning* | 73.3 (20.0) | 86.7 (20.0) | 0.111 |  |
| *Eating Problems* | 100.0 (11.1) | 100.0 (0.0) | 0.257 |  |
| *Treatment Burden* | 66.7 (33.3) | 88.9 (38.9) | 0.010 |  |
| *Health Perceptions* | 77.8 (22.2) | 88.9 (22.2) | 0.019 |  |
| *Social/School Functioning* | 66.7 (33.3) | 88.9 (27.8) | 0.033 |  |
| *Body Image* | 77.8 (33.3) | 88.9 (22.2) | 0.041 |  |
| *Role Functioning* | 91.7 (16.7) | 100.0 (8.3) | 0.028 |  |
| *Weight* | 100.0 (0.0) | 100.0 (0.0) | 0.459 |  |
| *Respiratory Symptoms* | 94.4 (16.7) | 88.9 (16.7) | 0.844 |  |
| *Digestive Symptoms* | 77.8 (33.3) | 88.9 (16.7) | 0.184 |  |
|  | ***n* = 54** | ***n* = 17** |  |  |
| **EQ-5D-5L Total Scores** |  |  | 0.587 |  |
| *11111 n(%)* | 24 (44.4) | 9 (52.9) |  |  |
| *any other health state n(%)* | 30 (55.6) | 8 (47.1) |  |  |
| *EQ Visual Analogue Scale score (0-100)* | 81.0 (15.0) | 90 (17.0) | 0.082 |  |
| ***Having any problem in five dimensions (score* ≥*2) n(%)*** | | | | |
| *Mobility* | 10 (18.5) | 0 (0.0) | 0.104 |  |
| *Self-care* | 2 (3.7) | 0 (0.0) | 1.000 |  |
| *Usual Activities***^~^** | 9 (16.6) | 0 (0.0) | 0.102 |  |
| *Pain / Discomfort* | 19 (35.2) | 3 (17.6) | 0.235 |  |
| *Anxiety / Depression Severity* | 25 (46.3) | 6 (35.3) | 0.577 |  |
| ***Dimension scores (1-5)*** |  |  |  |  |
| *Mobility (Walking)* | 1.0 (0.0) | 1.0 (0.0) | 0.058 |  |
| *Self-care (washing & dressing)* | 1.0 (0.0) | 1.0 (0.0) | 0.424 |  |
| *Usual Activities (e.g., work, study, housework, family or leisure activities)* | 1.0 (0.0) | 1.0 (0.0) | 0.074 |  |
| *Pain / Discomfort* | 1.0 (1.0) | 1.0 (0.0) | 0.144 |  |
| *Anxiety / Depression Severity* | 1.0 (1.0) | 1.0 (1.0) | 0.343 |  |
|  |  |  |  |  |
| **PAC-SYM Score (0-4)** | 0.4 (0.7) | 0.3 (0.5) | 0.971 |  |
| ***Subscales*** |  |  |  |  |
| *Abdominal Symptoms* | 0.5 (1.0) | 0.3 (1.2) | 0.277 |  |
| *Rectal Symptoms* | 0.0 (0.3) | 0.0 (0.2) | 0.683 |  |
| *Stool Symptoms* | 0.2 (0.7) | 0.2 (0.6) | 0.994 |  |
| ***Constipation symptom details*** |  |  |  |  |
| *discomfort in stomach* | 1.0 (1.0) | 0.0 (1.0) | 0.206 |  |
| *pain in your stomach* | 0.0 (1.0) | 0.0 (1.0) | 0.822 |  |
| *bloating in your stomach* | 1.0 (2.0) | 1.0 (2.0) | 0.474 |  |
| *stomach cramps* | 0.0 (1.0) | 0.0 (0.0) | 0.403 |  |
| *painful bowel movements* | 0.0 (0.0) | 0.0 (0.0) | 0.854 |  |
| *rectal burning during / after a bowel movement* | 0.0 (0.0) | 0.0 (0.0) | 0.660 |  |
| *rectal bleeding or tearing during / after a bowel movement* | 0.0 (0.0) | 0.0 (0.0) | 0.362 |  |
| *incomplete bowel movement, felt like you didn't finish* | 0.0 (1.0) | 0.5 (1.0) | 0.767 |  |
| *bowel movements were too hard* | 0.0 (0.3) | 0.0 (0.0) | 0.452 |  |
| *bowel movements were too small* | 0.0 (1.0) | 0.0 (1.0) | 0.420 |  |
| *straining or squeezing to try to pass bowel movements* | 0.0 (1.0) | 0.0 (0.0) | 0.398 |  |
| *feeling like you had to pass a bowel movement but you could not ('false alarm')* | 0.0 (0.0) | 0.0 (0.8) | 0.555 |  |
|  | ***n* = 55** | ***n* = 15** |  |  |
| **PAGI-SYM Score (0-5)** | 0.4 (0.6) | 0.2 (0.3) | 0.015 |  |
| ***Subscales*** |  |  |  |  |
| *Heartburn / Regurgitation* | 0.1 (0.3) | 0.0 (0.0) | 0.005 |  |
| *Nausea / Vomiting* | 0.0 (0.3) | 0.0 (0.0) | 0.436 |  |
| *Fullness / Satiety* | 0.5 (1.0) | 0.3 (0.8) | 0.124 |  |
| *Bloating* | 1.0 (2.0) | 0.5 (1.0) | 0.063 |  |
| *Upper-Abdominal Pain* | 0.0 (0.5) | 0.0 (0.0) | 0.019 |  |
| *Lower-Abdominal Pain* | 0.0 (0.5) | 0.0 (0.0) | 0.127 |  |
| ***Gastrointestinal symptom details*** |  |  |  |  |
| *Nausea* | 0.0 (1.0) | 0.0 (0.0) | 0.643 |  |
| *Retching* | 0.0 (0.0) | 0.0 (0.0) | 0.732 |  |
| *Vomiting* | 0.0 (0.0) | 0.0 (0.0) | 0.354 |  |
| *Stomach fullness* | 1.0 (2.0) | 0.0 (2.0) | 0.294 |  |
| *Not able to finish a normal sized meal* | 0.0 (1.0) | 0.0 (0.0) | 0.157 |  |
| *Feeling excessively full after meals* | 0.0 (1.5) | 0.0 (1.0) | 0.307 |  |
| *Loss of appetite* | 1.0 (1.0) | 0.0 (0.0) | 0.651 |  |
| *Bloating* | 1.0 (2.0) | 0.0 (1.0) | 0.128 |  |
| *Stomach or belly visibly larger* | 1.0 (2.0) | 0.0 (1.0) | 0.090 |  |
| *Upper abdominal pain* | 0.0 (0.0) | 0.0 (0.0) | 0.093 |  |
| *Upper abdominal discomfort* | 0.0 (0.3) | 0.0 (0.0) | 0.037 |  |
| *Lower abdominal pain* | 0.0 (0.0) | 0.0 (0.0) | 0.683 |  |
| *Lower abdominal discomfort* | 0.0 (1.0) | 0.0 (0.0) | 0.199 |  |
| *Heartburn during the day* | 0.0 (1.0) | 0.0 (0.0) | 0.102 |  |
| *Heartburn when lying down* | 0.0 (0.3) | 0.0 (0.0) | 0.390 |  |
| *Discomfort inside your chest during the day* | 0.0 (0.0) | 0.0 (0.0) | 0.487 |  |
| *Discomfort inside your chest at night* | 0.0 (0.0) | 0.0 (0.0) | 0.145 |  |
| *Regurgitation or reflux during the day* | 0.0 (1.0) | 0.0 (0.0) | 0.102 |  |
| *Regurgitation or reflux when lying down* | 0.0 (0.0) | 0.0 (0.0) | 0.047 |  |
| *Bitter, acid or sour taste in your mouth* | 0.0 (0.0) | 0.0 (0.0) | 0.074 |  |
| Values are represented as median (interquartile range) or *n* (%).  *P*-values were derived with chi-square tests, Mann Whitney U and Kruskal-Wallis tests (non-parametric).  Significance derived from a *p*-value of <0.05.  **^~^**Work, study, housework, family or leisure activities.  Abbreviations: PROM Patient-reported outcome measure, PI, Pancreatic Insufficient, PS, Pancreatic Sufficient, CFQ-R Cystic Fibrosis Questionnaire – Revised, EQ-5D-5L EuroQol 5-dimension 5-level questionnaire, EQ EuroQol, PAC-SYM Patient Assessment of Constipation Symptoms questionnaire, PAGI-SYM Patient Assessment of Upper Gastrointestinal Symptoms questionnaire. | | | |  |

Table S4. PROMs (CFQ-R, EQ-5D-5L, PAC-SYM and PAGI-SYM) in adults living with CF by those above or below the mean FEV_1_%.

| **PROM** | **Below Mean FEV_1_% (*n* = 30)** | **Above Mean FEV_1_% (*n* = 42)** | ***p*-values** |  |
| --- | --- | --- | --- | --- |
| **CFQ-R Domain Scores (0-100)** |  |  |  |  |
| *Physical Functioning* | 85.4 (38.5) | 95.8 (21.9) | 0.009 |  |
| *Vitality* | 66.7 (29.2) | 66.7 (25.0) | 0.571 |  |
| *Emotional Functioning* | 76.7 (23.3) | 76.7 (20.0) | 0.769 |  |
| *Eating Problems* | 100.0 (0.0) | 100.0 (11.1) | 0.144 |  |
| *Treatment Burden* | 66.7 (33.3) | 77.8 (33.3) | 0.754 |  |
| *Health Perceptions* | 77.8 (16.7) | 88.9 (33.3) | 0.035 |  |
| *Social/School Functioning* | 66.7 (24.7) | 77.8 (27.8) | 0.129 |  |
| *Body Image* | 77.8 (36.1) | 88.9 (44.5) | 0.641 |  |
| *Role Functioning* | 91.7 (16.7) | 91.7 (16.7) | 0.871 |  |
| *Weight* | 100.0 (0.0) | 100.0 (0.0) | 0.557 |  |
| *Respiratory Symptoms* | 83.3 (12.5) | 94.4 (16.7) | 0.055 |  |
| *Digestive Symptoms* | 88.9 (33.3) | 77.8 (25.0) | 0.123 |  |
|  | ***n* = 29** |  |  |  |
| **EQ-5D-5L Total Scores** |  |  | 0.480 |  |
| *11111 n(%)* | 24 (44.4) | 9 (52.9) |  |  |
| *any other health state n(%)* | 30 (55.6) | 8 (47.1) |  |  |
| *EQ Visual Analogue Scale score (0-100)* | 81.0 (21.0) | 85.0 (10.0) | 0.224 |  |
| ***Having any problem in five dimensions (score* ≥*2) n(%)*** | | | | |
| *Mobility* | 10 (18.5) | 0 (0.0) | 0.079 |  |
| *Self-care* | 2 (3.7) | 0 (0.0) | 0.163 |  |
| *Usual Activities***^~^** | 9 (16.6) | 0 (0.0) | 0.471 |  |
| *Pain / Discomfort* | 19 (35.2) | 3 (17.6) | 0.434 |  |
| *Anxiety / Depression Severity* | 25 (46.3) | 6 (35.3) | 0.811 |  |
| ***Dimension scores (1-5)*** |  |  |  |  |
| *Mobility (Walking)* | 1.0 (0.5) | 1.0 (0.0) | 0.045 |  |
| *Self-care (washing & dressing)* | 1.0 (0.0) | 1.0 (0.0) | 0.086 |  |
| *Usual Activities***^~^** | 1.0 (0.0) | 1.0 (0.0) | 0.341 |  |
| *Pain / Discomfort* | 1.0 (0.5) | 1.0 (1.0) | 0.352 |  |
| *Anxiety / Depression Severity* | 1.0 (1.0) | 1.0 (1.0) | 0.713 |  |
|  | ***n* = 30** | ***n* = 41** |  |  |
| **PAC-SYM Score (0-4)** | 0.3 (0.7) | 0.4 (0.7) | 0.374 |  |
| ***Subscales*** |  |  |  |  |
| *Abdominal Symptoms* | 0.3 (0.9) | 0.5 (1.1) | 0.078 |  |
| *Rectal Symptoms* | 0.0 (0.3) | 0.0 (0.3) | 0.831 |  |
| *Stool Symptoms* | 0.1 (0.6) | 0.2 (0.8) | 0.741 |  |
| ***Constipation symptom details*** |  |  |  |  |
| *discomfort in stomach* | 0.0 (1.0) | 1.0 (2.0) | 0.014 |  |
| *pain in your stomach* | 0.0 (1.0) | 0.0 (1.0) | 0.145 |  |
| *bloating in your stomach* | 1.0 (2.0) | 1.0 (2.0) | 0.501 |  |
| *stomach cramps* | 0.0 (0.0) | 0.0 (1.0) | 0.208 |  |
| *painful bowel movements* | 0.0 (0.0) | 0.0 (0.5) | 0.432 |  |
| *rectal burning during / after a bowel movement* | 0.0 (0.0) | 0.0 (0.0) | 0.964 |  |
| *rectal bleeding or tearing during / after a bowel movement* | 0.0 (0.0) | 0.0 (0.0) | 0.576 |  |
| *incomplete bowel movement, felt like you didn't finish* | 0.0 (1.0) | 0.0 (1.0) | 0.508 |  |
| *bowel movements were too hard* | 0.0 (0.0) | 0.0 (0.0) | 0.791 |  |
| *bowel movements were too small* | 0.0 (1.0) | 0.0 (1.0) | 0.612 |  |
| *straining or squeezing to try to pass bowel movements* | 0.0 (1.0) | 0.0 (0.8) | 0.455 |  |
| *feeling like you had to pass a bowel movement but you could not ('false alarm')* | 0.0 (0.0) | 0.0 (0.0) | 0.620 |  |
|  | ***n* = 30** | ***n* = 39** |  |  |
| **PAGI-SYM Score (0-5)** | 0.3 (0.6) | 0.3 (0.6) | 0.729 |  |
| ***Subscales*** |  |  |  |  |
| *Heartburn / Regurgitation* | 0.1 (0.3) | 0.1 (0.3) | 0.924 |  |
| *Nausea / Vomiting* | 0.0 (0.0) | 0.0 (0.3) | 0.248 |  |
| *Fullness / Satiety* | 0.4 (1.0) | 0.5 (1.0) | 0.971 |  |
| *Bloating* | 1.0 (2.1) | 1.0 (2.0) | 0.995 |  |
| *Upper-Abdominal Pain* | 0.0 (0.5) | 0.0 (0.0) | 0.376 |  |
| *Lower-Abdominal Pain* | 0.0 (0.5) | 0.0 (0.5) | 0.756 |  |
| ***Gastrointestinal symptom details*** |  |  |  |  |
| *Nausea* | 0.0 (0.0) | 0.0 (1.0) | 0.270 |  |
| *Retching* | 0.0 (0.0) | 0.0 (0.0) | 0.738 |  |
| *Vomiting* | 0.0 (0.0) | 0.0 (0.0) | 0.123 |  |
| *Stomach fullness* | 1.0 (2.0) | 1.0 (2.0) | 0.485 |  |
| *Not able to finish a normal sized meal* | 0.0 (0.0) | 0.0 (1.0) | 0.362 |  |
| *Feeling excessively full after meals* | 0.0 (1.0) | 0.0 (1.0) | 0.564 |  |
| *Loss of appetite* | 0.0 (0.3) | 0.0 (1.0) | 0.755 |  |
| *Bloating* | 1.0 (2.0) | 1.0 (2.0) | 0.719 |  |
| *Stomach or belly visibly larger* | 1.0 (2.3) | 1.0 (2.0) | 0.975 |  |
| *Upper abdominal pain* | 0.0 (0.0) | 0.0 (0.0) | 0.159 |  |
| *Upper abdominal discomfort* | 0.0 (0.0) | 0.0 (0.0) | 0.790 |  |
| *Lower abdominal pain* | 0.0 (0.0) | 0.0 (0.0) | 0.474 |  |
| *Lower abdominal discomfort* | 0.0 (1.0) | 0.0 (1.0) | 0.823 |  |
| *Heartburn during the day* | 0.0 (0.0) | 0.0 (1.0) | 0.248 |  |
| *Heartburn when lying down* | 0.0 (1.0) | 0.0 (0.0) | 0.083 |  |
| *Discomfort inside your chest during the day* | 0.0 (0.0) | 0.0 (0.0) | 0.831 |  |
| *Discomfort inside your chest at night* | 0.0 (0.0) | 0.0 (0.0) | 0.972 |  |
| *Regurgitation or reflux during the day* | 0.0 (0.0) | 0.0 (1.0) | 0.294 |  |
| *Regurgitation or reflux when lying down* | 0.0 (0.3) | 0.0 (0.0) | 0.240 |  |
| *Bitter, acid or sour taste in your mouth* | 0.0 (0.0) | 0.0 (0.0) | 0.313 |  |
| Values are represented as median (interquartile range) or *n* (%).  *P*-values were derived with chi-square tests, Mann Whitney U and Kruskal-Wallis tests (non-parametric).  Significance derived from a *p*-value of <0.05.  **^~^**Work, study, housework, family or leisure activities.  Abbreviations: PROM Patient-reported outcome measure, FEV1%, Predicted Percentage Forced Expiratory Volume, CFQ-R Cystic Fibrosis Questionnaire – Revised, EQ-5D-5L EuroQol 5-dimension 5-level questionnaire, EQ EuroQol, PAC-SYM Patient Assessment of Constipation Symptoms questionnaire, PAGI-SYM Patient Assessment of Upper Gastrointestinal Symptoms questionnaire. | | | |  |

Table S5. PROMs (CFQ-R, EQ-5D-5L, PAC-SYM and PAGI-SYM) in adults living with CF by modulator use.

| **PROM** | **Uses modulators**  **(*n* = 57)** | **Not making modulators**  **(*n* = 15)** | ***p*-values** |  |
| --- | --- | --- | --- | --- |
| **CFQ-R Domain Scores (0-100)** |  |  |  |  |
| *Physical Functioning* | 87.5 (35.4) | 95.8 (16.7) | 0.164 |  |
| *Vitality* | 66.7 (25.0) | 66.7 (25.0) | 0.653 |  |
| *Emotional Functioning* | 73.3 (23.3) | 80.0 (20.0) | 0.649 |  |
| *Eating Problems* | 100.0 (11.1) | 100.0 (11.1) | 0.628 |  |
| *Treatment Burden* | 77.8 (33.3) | 77.8 (33.3) | 0.307 |  |
| *Health Perceptions* | 77.8 (22.2) | 77.8 (22.2) | 0.214 |  |
| *Social/School Functioning* | 66.7 (33.3) | 94.4 (27.8) | 0.011 |  |
| *Body Image* | 77.8 (38.9 | 77.8 (44.5) | 0.729 |  |
| *Role Functioning* | 91.7 (16.7) | 100.0 (16.7) | 0.091 |  |
| *Weight* | 100.0 (0.0) | 100.0 (33.3) | 0.142 |  |
| *Respiratory Symptoms* | 88.9 (16.7) | 94.4 (16.7) | 0.625 |  |
| *Digestive Symptoms* | 77.8 (33.3) | 88.9 (33.3) | 0.269 |  |
|  | ***n* = 56** |  |  |  |
| **EQ-5D-5L Total Scores** |  |  | 0.260 |  |
| *11111 n(%)* | 24 (44.4) | 9 (52.9) |  |  |
| *any other health state n(%)* | 30 (55.6) | 8 (47.1) |  |  |
| *EQ Visual Analogue Scale score (0-100)* | 84.0 (15.0) | 81.0 (15.0) | 0.260 |  |
| ***Having any problem in five dimensions (score* ≥*2) n(%)*** | | | | |
| *Mobility* | 10 (18.5) | 0 (0.0) | 0.106 |  |
| *Self-care* | 2 (3.7) | 0 (0.0) | 1.000 |  |
| *Usual Activities***^~^** | 9 (16.6) | 0 (0.0) | 1.000 |  |
| *Pain / Discomfort* | 19 (35.2) | 3 (17.6) | 0.027 |  |
| *Anxiety / Depression Severity* | 25 (46.3) | 6 (35.3) | 0.398 |  |
| ***Dimension scores (1-5)*** |  |  |  |  |
| *Mobility (Walking)* | 1.0 (0.0) | 1.0 (0.0) | 0.080 |  |
| *Self-care (washing & dressing)* | 1.0 (0.0) | 1.0 (0.0) | 0.461 |  |
| *Usual Activities***^~^** | 1.0 (0.0) | 1.0 (0.0) | 0.971 |  |
| *Pain / Discomfort* | 1.0 (1.0) | 1.0 (0.0) | 0.022 |  |
| *Anxiety / Depression Severity* | 1.0 (1.0) | 1.0 (1.0) | 0.219 |  |
|  | ***n* = 57** | ***n* = 14** |  |  |
| **PAC-SYM Score (0-4)** | 0.4 (0.7) | 0.3 (0.6) | 0.374 |  |
| ***Subscales*** |  |  |  |  |
| *Abdominal Symptoms* | 0.5 (1.3) | 0.6 (0.8) | 0.775 |  |
| *Rectal Symptoms* | 0.0 (0.3) | 0.0 (0.3) | 0.459 |  |
| *Stool Symptoms* | 0.2 (0.8) | 0.2 (0.4) | 0.723 |  |
| ***Constipation symptom details*** |  |  |  |  |
| *discomfort in stomach* | 1.0 (1.0) | 0.5 (1.0) | 0.662 |  |
| *pain in your stomach* | 0.0 (1.0) | 0.0 (1.0) | 0.648 |  |
| *bloating in your stomach* | 1.0 (2.0) | 1.0 (1.3) | 0.758 |  |
| *stomach cramps* | 0.0 (1.0) | 0.0 (0.3) | 0.390 |  |
| *painful bowel movements* | 0.0 (0.0) | 0.0 (0.3) | 0.951 |  |
| *rectal burning during / after a bowel movement* | 0.0 (0.0) | 0.0 (0.0) | 0.171 |  |
| *rectal bleeding or tearing during / after a bowel movement* | 0.0 (0.0) | 0.0 (0.0) | 0.389 |  |
| *incomplete bowel movement, felt like you didn't finish* | 0.0 (1.0) | 0.5 (1.0) | 0.892 |  |
| *bowel movements were too hard* | 0.0 (0.8) | 0.0 (0.0) | 0.179 |  |
| *bowel movements were too small* | 0.0 (1.0) | 0.0 (1.0) | 0.572 |  |
| *straining or squeezing to try to pass bowel movements* | 0.0 (1.0) | 0.0 (0.0) | 0.009 |  |
| *feeling like you had to pass a bowel movement but you could not ('false alarm')* | 0.0 (0.0) | 0.0 (0.3) | 0.839 |  |
|  | ***n* = 56** | ***n* = 13** |  |  |
| **PAGI-SYM Score (0-5)** | 0.3 (0.6) | 0.2 (0.6) | 0.326 |  |
| ***Subscales*** |  |  |  |  |
| *Heartburn / Regurgitation* | 0.1 (0.3) | 0.0 (0.3) | 0.361 |  |
| *Nausea / Vomiting* | 0.0 (0.3) | 0.0 (0.0) | 0.073 |  |
| *Fullness / Satiety* | 0.5 (1.0) | 0.3 (0.9) | 0.350 |  |
| *Bloating* | 1.0 (2.0) | 1.0 (1.8) | 0.735 |  |
| *Upper-Abdominal Pain* | 0.0 (0.0) | 0.0 (0.0) | 0.499 |  |
| *Lower-Abdominal Pain* | 0.0 (0.5) | 0.0 ().3) | 0.279 |  |
| ***Gastrointestinal symptom details*** |  |  |  |  |
| *Nausea* | 0.0 (1.0) | 0.0 (0.0) | 0.122 |  |
| *Retching* | 0.0 (0.0) | 0.0 (0.0) | 0.221 |  |
| *Vomiting* | 0.0 (0.0) | 0.0 (0.0) | 0.397 |  |
| *Stomach fullness* | 1.0 (2.0) | 0.0 (1.5) | 0.275 |  |
| *Not able to finish a normal sized meal* | 0.0 (0.8) | 0.0 (1.0) | 0.676 |  |
| *Feeling excessively full after meals* | 0.0 (1.0) | 0.0 (1.0) | 0.542 |  |
| *Loss of appetite* | 0.0 (0.8) | 0.0 (0.5) | 0.968 |  |
| *Bloating* | 1.0 (2.0) | 1.0 (2.0) | 0.804 |  |
| *Stomach or belly visibly larger* | 1.0 (2.0) | 0.0 (2.0) | 0.489 |  |
| *Upper abdominal pain* | 0.0 (0.0) | 0.0 (0.0) | 0.773 |  |
| *Upper abdominal discomfort* | 0.0 (0.0) | 0.0 (0.0) | 0.669 |  |
| *Lower abdominal pain* | 0.0 (0.0) | 0.0 (0.0) | 0.290 |  |
| *Lower abdominal discomfort* | 0.0 (1.0) | 0.0 (0.5) | 0.394 |  |
| *Heartburn during the day* | 0.0 (1.0) | 0.0 (0.0) | 0.129 |  |
| *Heartburn when lying down* | 0.0 (0.0) | 0.0 (0.0) | 0.475 |  |
| *Discomfort inside your chest during the day* | 0.0 (0.0) | 0.0 (0.0) | 0.057 |  |
| *Discomfort inside your chest at night* | 0.0 (0.0) | 0.0 (0.0) | 0.183 |  |
| *Regurgitation or reflux during the day* | 0.0 (0.0) | 0.0 (0.5) | 0.901 |  |
| *Regurgitation or reflux when lying down* | 0.0 (0.0) | 0.0 (0.0) | 0.727 |  |
| *Bitter, acid or sour taste in your mouth* | 0.0 (0.0) | 0.0 (0.0) | 0.880 |  |
| Values are represented as median (interquartile range) or *n* (%).  *P*-values were derived with chi-square tests, Mann Whitney U and Kruskal-Wallis tests (non-parametric).  Significance derived from a *p*-value of <0.05.  **^~^**Work, study, housework, family or leisure activities.  Abbreviations: PROM Patient-reported outcome measure, CFQ-R Cystic Fibrosis Questionnaire – Revised, EQ-5D-5L EuroQol 5-dimension 5-level questionnaire, EQ EuroQol, PAC-SYM Patient Assessment of Constipation Symptoms questionnaire, PAGI-SYM Patient Assessment of Upper Gastrointestinal Symptoms questionnaire. | | | |  |

Table S6. PROMs (CFQ-R, EQ-5D-5L, PAC-SYM and PAGI-SYM) in adults living with CF by income status.

| **PROM** | **Full-Income**  **(*n* = 33)** | **Part-time Income (*n* = 14)** | **No Income**  **(*n* = 25)** | ***p*-values** | |
| --- | --- | --- | --- | --- | --- |
| **CFQ-R Domain Scores (0-100)** |  |  |  |  | |
| *Physical Functioning* | 95.8 (16.7) | 89.6 (49.0) | 70.8 (39.6) | 0.088 | |
| *Vitality* | 75.0 (16.7) | 50.0 (35.4) | 66.7 (29.2) | 0.027 | |
| *Emotional Functioning* | 80.0 (16.7) | 60.0 (31.7) | 80.0 (23.3) | 0.012 | |
| *Eating Problems* | 100.0 (0.0) | 94.4 (22.2) | 100.0 (11.1) | 0.069 | |
| *Treatment Burden* | 77.8 (33.3) | 55.6 (33.3) | 66.7 (33.3) | 0.031 | |
| *Health Perceptions* | 88.9 (33.3) | 77.8 (13.9) | 77.8 (22.2) | 0.123 | |
| *Social/School Functioning* | 83.3 (27.8) | 63.9 (38.9) | 66.7 (36.1) | 0.047 | |
| *Body Image* | 88.9 (22.2) | 55.6 (55.6) | 77.8 (33.3) | 0.003 | |
| *Role Functioning* | 100.0 (8.3) | 83.3 (33.3) | 91.7 (8.3) | 0.010 | |
| *Weight* | 100.0 (0.0) | 100.0 (41.7) | 100.0 (0.0) | 0.207 | |
| *Respiratory Symptoms* | 94.4 (16.7) | 83.3 (23.6) | 88.9 (11.1) | 0.079 | |
| *Digestive Symptoms* | 88.9 (27.8) | 66.7 (36.1) | 88.9 (33.3) | 0.184 | |
|  | ***n* = 33** | ***n* = 14** | ***n* = 24** |  | |
| **EQ-5D-5L Total Scores** |  |  |  | 0.453 | |
| *11111 n(%)* | 18 (54.5) | 4 (28.6) | 11 (45.8) |  | |
| *any other health state n(%)* | 15 (30.3) | 10 (71.4) | 13 (54.2) |  | |
| *EQ Visual Analogue Scale score (0-100)* | 85.0 (20.0) | 88.0 (25.0) | 81.0 (11.0) | 0.345 | |
| ***Having any problem in five dimensions (score* ≥*2) n(%)*** | | | | |  |
| *Mobility* | 1 (3.0) | 3 (21.4) | 6 (25.0) | 0.029 | |
| *Self-care* | 0 (0.0) | 0 (0.0) | 2 (8.3) | 0.148 | |
| *Usual Activities***^~^** | 2 (6.1) | 2 (14.3) | 5 (20.8) | 0.241 | |
| *Pain / Discomfort* | 8 (24.2) | 6 (42.9) | 8 (33.3) | 0.469 | |
| *Anxiety / Depression Severity* | 11 (33.3) | 9 (64.3) | 11 (45.8) | 0.293 | |
| ***Dimension scores (1-5)*** |  |  |  |  | |
| *Mobility (Walking)* | 1.0 (0.0) | 1.0 (0.3) | 1.0 (0.8) | 0.050 | |
| *Self-care (washing & dressing)* | 1.0 (0.0) | 1.0 (0.0) | 1.0 (0.0) | 0.137 | |
| *Usual Activities***^~^** | 1.0 (0.0) | 1.0 (0.0) | 1.0 (0.0) | 0.265 | |
| *Pain / Discomfort* | 1.0 (0.5) | 1.0 (1.0) | 1.0 (1.0) | 0.354 | |
| *Anxiety / Depression Severity* | 1.0 (1.0) | 2.0 (2.0) | 1.0 (1.0) | 0.110 | |
|  | ***n* = 32** |  | ***n* = 25** |  | |
| **PAC-SYM Score (0-4)** | 0.3 (0.5) | 0.6 (0.7) | 0.3 (0.8) | 0.110 | |
| ***Subscales*** |  |  |  |  | |
| *Abdominal Symptoms* | 0.5 (0.8) | 1.1 (1.1) | 0.3 (1.6) | 0.087 | |
| *Rectal Symptoms* | 0.0 (0.0) | 0.0 (0.3) | 0.0 (0.3) | 0.054 | |
| *Stool Symptoms* | 0.2 (0.6) | 0.2 (0.7) | 0.0 (0.6) | 0.301 | |
| ***Constipation symptom details*** |  |  |  |  | |
| *discomfort in stomach* | 0.5 (1.0) | 1.0 (2.0) | 0.0 (2.0) | 0.355 | |
| *pain in your stomach* | 0.0 (1.0) | 1.0 (1.0) | 0.0 (1.0) | 0.116 | |
| *bloating in your stomach* | 1.0 (2.0) | 2.0 (1.0) | 1.0 (2.5) | 0.097 | |
| *stomach cramps* | 0.0 (0.0) | 0.5 (1.3) | 0.0 (1.0) | 0.090 | |
| *painful bowel movements* | 0.0 (0.0) | 0.0 (1.0) | 0.0 (0.0) | 0.236 | |
| *rectal burning during / after a bowel movement* | 0.0 (0.0) | 0.0 (0.3) | 0.0 (0.0) | 0.037 | |
| *rectal bleeding or tearing during / after a bowel movement* | 0.0 (0.0) | 0.0 (1.0) | 0.0 (0.0) | 0.181 | |
| *incomplete bowel movement, felt like you didn't finish* | 0.5 (1.0) | 1.0 (2.0) | 0.0 (1.0) | 0.250 | |
| *bowel movements were too hard* | 0.0 (0.0) | 0.0 (0.5) | 0.0 (0.0) | 0.949 | |
| *bowel movements were too small* | 0.0 (1.0) | 0.0 (1.0) | 0.0 (0.5) | 0.677 | |
| *straining or squeezing to try to pass bowel movements* | 0.0 (0.8) | 1.0 (1.0) | 0.0 (0.0) | 0.067 | |
| *feeling like you had to pass a bowel movement but you could not ('false alarm')* | 0.0 (0.0) | 0.0 (1.0) | 0.0 (0.0) | 0.449 | |
|  |  | ***n* = 13** | ***n* = 24** |  | |
| **PAGI-SYM Score (0-5)** | 0.2 (0.4) | 0.7 (0.9) | 0.3 (1.2) | 0.022 | |
| ***Subscales*** |  |  |  |  | |
| *Heartburn / Regurgitation* | 0.0 (0.1) | 0.3 (1.1) | 0.0 (0.4) | 0.002 | |
| *Nausea / Vomiting* | 0.0 (0.0) | 0.3 (0.3) | 0.0 (0.3) | 0.080 | |
| *Fullness / Satiety* | 0.3 (0.8) | 0.5 (0.6) | 0.4 (1.2) | 0.083 | |
| *Bloating* | 0.8 (1.0) | 2.0 (2.0) | 0.8 (2.5) | 0.018 | |
| *Upper-Abdominal Pain* | 0.0 (0.0) | 0.0 (0.8) | 0.0 (0.4) | 0.348 | |
| *Lower-Abdominal Pain* | 0.0 (0.4) | 0.0 (1.0) | 0.0 (0.9) | 0.157 | |
| ***Gastrointestinal symptom details*** |  |  |  |  | |
| *Nausea* | 0.0 (0.0) | 0.0 (1.0) | 0.0 (1.0) | 0.120 | |
| *Retching* | 0.0 (0.0) | 0.0 (0.0) | 0.0 (0.0) | 0.195 | |
| *Vomiting* | 0.0 (0.0) | 0.0 (0.0) | 0.0 (0.0) | 0.429 | |
| *Stomach fullness* | 1.0 (1.8) | 1.0 (1.0) | 1.0 (2.0) | 0.265 | |
| *Not able to finish a normal sized meal* | 0.0 (0.0) | 1.0 (2.0) | 0.0 (0.0) | 0.052 | |
| *Feeling excessively full after meals* | 0.0 (1.0) | 0.0 (2.0) | 0.0 (2.0) | 0.577 | |
| *Loss of appetite* | 0.0 (0.0) | 0.0 (1.0) | 0.0 (1.0) | 0.264 | |
| *Bloating* | 1.0 (1.8) | 2.0 (2.0) | 1.0 (2.0) | 0.084 | |
| *Stomach or belly visibly larger* | 0.5 (1.0) | 2.0 (2.0) | 1.0 (3.0) | 0.015 | |
| *Upper abdominal pain* | 0.0 (0.0) | 0.0 (0.5) | 0.0 (0.0) | 0.239 | |
| *Upper abdominal discomfort* | 0.0 (0.0) | 0.0 (1.0) | 0.0 (0.0) | 0.349 | |
| *Lower abdominal pain* | 0.0 (0.0) | 0.0 (1.0) | 0.0 (0.0) | 0.160 | |
| *Lower abdominal discomfort* | 0.0 (0.0) | 0.0 (1.5) | 0.0 (1.0) | 0.141 | |
| *Heartburn during the day* | 0.0 (0.0) | 0.0 (2.0) | 0.0 (0.8) | 0.030 | |
| *Heartburn when lying down* | 0.0 (0.0 | 1.0 (2.0) | 0.0 (0.8) | 0.001 | |
| *Discomfort inside your chest during the day* | 0.0 (0.0) | 0.0 (1.0) | 0.0 (0.0) | 0.014 | |
| *Discomfort inside your chest at night* | 0.0 (0.0) | 0.0 (0.5) | 0.0 (0.0) | 0.194 | |
| *Regurgitation or reflux during the day* | 0.0 (0.0) | 0.0 (1.5) | 0.0 (1.0) | 0.282 | |
| *Regurgitation or reflux when lying down* | 0.0 (0.0) | 0.0 (1.0) | 0.0 (0.0) | 0.295 | |
| *Bitter, acid or sour taste in your mouth* | 0.0 (0.0) | 0.0 (1.0) | 0.0 (0.0) | 0.025 | |
| Values are represented as median (interquartile range) or *n* (%).  *P*-values were derived with chi-square tests, Mann Whitney U and Kruskal-Wallis tests (non-parametric).  Significance derived from a *p*-value of <0.05.  **^~^**Work, study, housework, family or leisure activities.  Abbreviations: PROM Patient-reported outcome measure, CFQ-R Cystic Fibrosis Questionnaire – Revised, EQ-5D-5L EuroQol 5-dimension 5-level questionnaire, EQ EuroQol, PAC-SYM Patient Assessment of Constipation Symptoms questionnaire, PAGI-SYM Patient Assessment of Upper Gastrointestinal Symptoms questionnaire. | | | | | |
