# Supplementary material for: Interrelationships between diet quality and health-related quality of life in Irish adults living with cystic fibrosis
Source: Eur J Nutr. 2025 Jul 24;64(6):248. doi: 10.1007/s00394-025-03766-y (PMC12289792; doi:10.1007/s00394-025-03766-y)
Supplement: Supplementary file 2 — Supplementary Material 2 [file 394_2025_3766_MOESM2_ESM.docx]

## S2 DQI-I subgroup analysis

Participants with a FEV_1_% above the mean value had significantly higher DQI-I (53.6 ± 10.1, *p* = 0.026) diet quality scores compared to participants below the mean FEV_1_% (DQI-I: 48.3 ± 8.7). Participants above the mean FEV1% had significantly higher DQI-I component scores of fruits (3.0 (4.0), *p* = 0.023) and fibre (5.0 (2.0). *p* = 0.018) adequacy compared to participants below the mean FEV_1_% value (fruits adequacy: 1.0 (3.0); fibre adequacy: 3.0 (2.0)) (Table S7). Male participants had significantly higher DQI-I component scores for iron (5.0 (2.0), *p* = 0.000) and calcium (5.0 (0.0), *p* = 0.034) compared to female participants (iron: 3.0 (2.0); calcium: 5.0 (2.0)) (Table S8). Participants with a BMI classed as overweight or obese had significantly lower DQI-I empty calorie component scores (0.0 (0.0), *p* = 0.036) compared to participants with a BMI classed as underweight or normal (empty calories: 0.0 (0.0)) (Table S9). No significant differences in DQI-I aggregated or component scores were observed between pancreatic sufficient and insufficient participants (Table S10). Individuals taking modulators had significantly different DQI-I within-group variety (5.0 (2.0), *p* = 0.005) and iron adequacy (5.0 (2.0), *p* = 0.042) component scores compared to individuals not using modulators (within-group variety: 3.0 (4.0); iron adequacy: 3.0 (4.0)) (Table S11). No significant differences were observed in relation to DQI-I aggregated or component scores between individuals with different income status (data not shown).

Table S7. DQI-I diet quality score and associated component scores in Irish adults with CF who participated in this cross-sectional study below and above the mean FEV_1_% for the cohort.

| **Diet Quality Measure** | **All (*n* = 68)** | **Below Mean FEV_1_%**  **(*n* = 29)** | **Above Mean FEV_1_%**  **(*n* = 38)** | ***p*-value** |
| --- | --- | --- | --- | --- |
| **DQI-I Score (0-100)** | 51.2 ± 9.8 | 48.3 ± 8.7 | 53.6 ± 10.1 | 0.026 |
| **DQI-I Variety Component Scores (0-20)*** | | | | |
| *Food Group Variety Score (0-15)* | 12.0 (6.0) | 12.0 (3.0) | 12.0 (3.8) | 0.061 |
| *Within-Group Variety Score (0-5)* | 3.0 (4.0) | 3.0 (4.0) | 3.0 (2.5) | 0.778 |
| **DQI-I Adequacy Component Scores (0-40)*** | | | | |
| *Vegetables (0-5)* | 3.0 (2.0) | 1.0 (2.0) | 3.0 (2.5) | 0.109 |
| *Fruits (0-5)* | 2.0 (4.0) | 1.0 (3.0) | 3.0 (4.0) | 0.023 |
| *Grains (0-5)* | 3.0 (2.0) | 3.0 (2.0) | 3.0 (2.0) | 0.907 |
| *Fibre (0-5)* | 5.0 (2.0) | 3.0 (2.0) | 5.0 (2.0) | 0.018 |
| *Protein (0-5)* | 5.0 (0.0) | 5.0 (0.0) | 5.0 (0.0) | 0.382 |
| *Iron (0-5)* | 3.0 (3.5) | 3.0 (2.0) | 3.0 (4.0) | 0.250 |
| *Calcium (0-5)* | 5.0 (2.0) | 5.0 (2.0) | 5.0 (2.0) | 0.189 |
| *Vitamin C (0-5)* | 5.0 (3.5) | 3.0 (4.0) | 5.0 (2.5) | 0.342 |
| **DQI-I Moderation Component Scores (0-30)*** | | | | |
| *Total Fat (0-6)* | 0.0 (0.0) | 0.0 (0.0) | 0.0 (0.0) | 0.397 |
| *Saturated Fat (0-6)* | 0.0 (0.0) | 0.0 (0.0) | 0.0 (0.0) | 0.397 |
| *Cholesterol (0-6)* | 6.0 (6.0) | 6.0 (6.0) | 6.0 (3.0) | 0.834 |
| *Sodium (0-6)* | 3.0 (5.3) | 3.0 (6.0) | 4.5 (3.0) | 0.059 |
| *Empty Calorie (0-6)* | 0.0 (0.0) | 0.0 (0.0) | 0.0 (0.0) | 0.582 |
| **DQI-I Overall Balance Scores (0-10)*** | | | | |
| *Macronutrient Ratio (0-6)* | 0.0 (0.0) | 0.0 (0.0) | 0.0 (0.0) | 0.724 |
| *Fatty Acid Ratio (0-4)* | 0.0 (0.0) | 0.0 (0.0) | 0.0 (0.0) | 0.719 |
| Parametric variables are presented as mean **±** SD and non-parametric variables as median (interquartile range).  *P*-values were derived with independent sample t-tests (Non-parametric: Mann Whitney U-test).  Significance was derived from a *p*-value of <0.05.  *Non-parametric test.  Mean FEV_1_%: 78.7 ± 24.9%.  Of participants who completed the three-day food diary, *n* = 1 did not report their FEV_1_%.  Abbreviations: FEV_1_%, forced expiratory volume as a percentage predicted; DQI-I, Diet Quality Index – International; SD, standard deviation. | | | | |

Table S8. DQI-I diet quality scores and associated component scores by gender/sex in Irish adults with CF.

|  | **Male (*n* = 28)** | | | **Female (*n* = 40)** | | | ***p*-value** | | |
| --- | --- | --- | --- | --- | --- | --- | --- | --- | --- |
| **DQI-I Score (0-100)** | 53.0 ± 9.7 | | | 50.0 ± 9.7 | | | 0.206 | | |
| **DQI-I Variety Component Scores (0-20)*** | |  |  | |  |  | |  |  |
| *Food Group Variety Score (0-15)* | 12.0 (5.3) | | | 12.0 (5.3) | | | 0.317 | | |
| *Within-Group Variety Score (0-5)* | 3.0 (2.0) | | | 3.0 (3.5) | | | 0.055 | | |
| **DQI-I Adequacy Component Scores (0-40)*** | |  |  | |  |  | |  |  |
| *Vegetables (0-5)* | 3.0 (2.0) | | | 3.0 (2.0) | | | 0.942 | | |
| *Fruits (0-5)* | 3.0 (4.0) | | | 1.0 (4.0) | | | 0.606 | | |
| *Grains (0-5)* | 3.0 (2.0) | | | 3.0 (2.0) | | | 0.426 | | |
| *Fibre (0-5)* | 5.0 (2.0) | | | 5.0 (2.0) | | | 0.644 | | |
| *Protein (0-5)* | 5.0 (0..0) | | | 5.0 (0.0) | | | 0.233 | | |
| *Iron (0-5)* | 5.0 (2.0) | | | 3.0 (2.0) | | | 0.000 | | |
| *Calcium (0-5)* | 5.0 (0.0) | | | 5.0 (2.0) | | | 0.034 | | |
| *Vitamin C (0-5)* | 5.0 (4.0) | | | 5.0 (2.0) | | | 0.827 | | |
| **DQI-I Moderation Component Scores (0-30)*** | |  |  | |  |  |  |  |  |
| *Total Fat (0-6)* | 0.0 (0.0) | | | 0.0 (0.0) | | | 0.953 | | |
| *Saturated Fat (0-6)* | 0.0 (0.0) | | | 0.0 (0.0) | | | 0.953 | | |
| *Cholesterol (0-6)* | 4.5 (6.0) | | | 6.0 (5.3) | | | 0.681 | | |
| *Sodium (0-6)* | 3.0 (5.3) | | | 3.0 (5.3) | | | 0.873 | | |
| *Empty Calorie (0-6)* | 0.0 (0.0) | | | 0.0 (0.0) | | | 0.259 | | |
| **DQI-I Overall Balance Scores (0-10)*** | | | | | | | | | |
| *Macronutrient Ratio (0-6)* | 0.0 (0.0) | | | 0.0 (0.0) | | | 0.779 | | |
| *Fatty Acid Ratio (0-4)* | 0.0 (1.5) | | | 0.0 (0.0) | | | 0.311 | | |
| Parametric variables are presented as mean **±** SD and non-parametric variables as median (interquartile range).  *P*-values were derived with independent sample t-tests (Non-parametric: Mann Whitney U-test).  Significance was derived from a *p*-value of <0.05.  *Non-parametric test.  Abbreviations: DQI-I, Diet Quality Index – International; SD, standard deviation. | | | | | | | | | |

Table S9. DQI-I diet quality scores and associated component scores by BMI in Irish adults with CF.

|  | **≤ 24.9 kg/m^2^**  **(*n* = 43)** | **≥ 25 kg/m^2~^**  **(*n* = 25)** | ***p*-value** | | |
| --- | --- | --- | --- | --- | --- |
| **DQI-I Score (0-100)** | 51.0 ± 9.2 | 51.7 ± 10.9 | 0.757 | |  |
| **DQI-I Variety Component Scores (0-20)*** | | | |  |  |
| *Food Group Variety Score (0-15)* | 12.0 (6.0) | 12.0 (3.0) | 0.506 | |  |
| *Within-Group Variety Score (0-5)* | 3.0 (4.0) | 3.0 (2.0) | 0.710 | |  |
| **DQI-I Adequacy Component Scores (0-40)*** | | | |  |  |
| *Vegetables (0-5)* | 3.0 (2.0) | 3.0 (2.0) | 0.357 | |  |
| *Fruits (0-5)* | 1.0 (5.0) | 3.0 (4.0) | 0.650 | |  |
| *Grains (0-5)* | 3.0 (2.0) | 3.0 (2.0) | 0.376 | |  |
| *Fibre (0-5)* | 5.0 (2.0) | 3.0 (2.0) | 0.189 | |  |
| *Protein (0-5)* | 5.0 (0.0) | 5.0 (0.0) | 0.277 | |  |
| *Iron (0-5)* | 3.0 (4.0) | 3.0 (3.0) | 0.541 | |  |
| *Calcium (0-5)* | 5.0 (2.0) | 5.0 (2.0) | 0.941 | |  |
| *Vitamin C (0-5)* | 5.0 (2.0) | 5.0 (4.0) | 0.972 | |  |
| **DQI-I Moderation Component Scores (0-30)*** | | | |  |  |
| *Total Fat (0-6)* | 0.0 (0.0) | 0.0 (0.0) | 0.053 | |  |
| *Saturated Fat (0-6)* | 0.0 (0.0) | 0.0 (0.0) | 0.053 | |  |
| *Cholesterol (0-6)* | 3.0 (6.0) | 6.0 (3.0) | 0.228 | |  |
| *Sodium (0-6)* | 3.0 (6.0) | 3.0 (4.5) | 0.704 | |  |
| *Empty Calorie (0-6)* | 0.0 (0.0) | 0.0 (0.0) | 0.036 | |  |
| **DQI-I Overall Balance Scores (0-10)*** | | | | |  |
| *Macronutrient Ratio (0-6)* | 0.0 (0.0) | 0.0 (0.0) | 0.275 | |  |
| *Fatty Acid Ratio (0-4)* | 0.0 (0.0) | 0.0 (0.0) | 0.867 | |  |
| Parametric variables are presented as mean **±** SD and non-parametric variables as median (interquartile range).  *P*-values were derived with independent sample t-tests (Non-parametric: Mann Whitney U-test).  Significance was derived from a *p*-value of <0.05.  *Non-parametric test.  **^~^**World Health Organisation cut-off values for individuals overweight/obese (44).  Abbreviations: DQI-I, Diet Quality Index – International; SD, standard deviation. | | | | |  |

Table S10. DQI-I diet quality scores and associated component scores by PI status in Irish adults with CF.

|  | **PI (*n* = 52)** | **PS (*n* = 15)** | ***p*-value** | |
| --- | --- | --- | --- | --- |
| **DQI-I Score (0-100)** | 51.6 ± 10.0 | 50.4 ± 9.4 | 0.928 |  |
| **DQI-I Variety Component Scores (0-20)*** | | | | |
| *Food Group Variety Score (0-15)* | 12.0 (6.0) | 12.0 (6.0) | 0.603 |  |
| *Within-Group Variety Score (0-5)* | 5.0 (4.0) | 3.0 (4.0) | 0.231 |  |
| **DQI-I Adequacy Component Scores (0-40)*** | | | | |
| *Vegetables (0-5)* | 3.0 (4.0) | 3.0 (2.0) | 0.444 |  |
| *Fruits (0-5)* | 3.0 (4.0) | 1.0 (5.0) | 0.214 |  |
| *Grains (0-5)* | 3.0 (2.0) | 3.0 (2.0) | 0.192 |  |
| *Fibre (0-5)* | 3.0 (2.0) | 5.0 (2.0) | 0.585 |  |
| *Protein (0-5)* | 5.0 (0.0) | 5.0 (0.0) | 0.529 |  |
| *Iron (0-5)* | 3.0 (4.0) | 3.0 (3.5) | 0.552 |  |
| *Calcium (0-5)* | 5.0 (2.0) | 5.0 (2.0) | 0.288 |  |
| *Vitamin C (0-5)* | 5.0 (2.0) | 3.0 (4.0) | 0.209 |  |
| **DQI-I Moderation Component Scores (0-30)*** | | | | |
| *Total Fat (0-6)* | 0.0 (0.0) | 0.0 (0.0) | 0.081 |  |
| *Saturated Fat (0-6)* | 0.0 (0.0) | 0.0 (0.0) | 0.081 |  |
| *Cholesterol (0-6)* | 6.0 (6.0) | 6.0 (5.3) | 0.794 |  |
| *Sodium (0-6)* | 3.0 (6.0) | 3.0 (3.0) | 0.911 |  |
| *Empty Calorie (0-6)* | 0.0 (0.0) | 0.0 (0.0) | 0.923 |  |
| **DQI-I Overall Balance Scores (0-10)*** | | | |  |
| *Macronutrient Ratio (0-6)* | 0.0 (0.0) | 0.0 (0.0) | 0.268 |  |
| *Fatty Acid Ratio (0-4)* | 0.0 (0.0) | 0.0 (0.0) | 0.236 |  |
| Parametric variables are presented as mean **±** SD and non-parametric variables as median (interquartile range).  *P*-values were derived with independent sample t-tests (Non-parametric: Mann Whitney U-test).  Significance was derived from a *p*-value of <0.05.  *Non-parametric test.  Of participants who completed the three-day food diary, *n* = 1 did not report PI / PS status.  Abbreviations: PI, pancreatic insufficiency; PS, pancreatic sufficiency; DQI-I, Diet Quality Index – International; SD, standard deviation. | | | | |

Table S11. DQI-I diet quality scores and associated component scores by modulator use in Irish adults with CF.

|  | **Not Taking Modulators**  **(*n* = 13)** | **Using Modulators**  **(*n* = 54)** | ***p-*value** | |  |
| --- | --- | --- | --- | --- | --- |
| **DQI-I Score (0-100)** | 54.2 ± 10.3 | 50.6 ± 9.7 | 0.233 |  |  |
| **DQI-I Variety Component Scores (0-20)*** | | | | | |
| *Food Group Variety Score (0-15)* | 12.0 (4.5) | 12.0 (6.0) | 0.442 |  |  |
| *Within-Group Variety Score (0-5)* | 5.0 (2.0) | 3.0 (4.0) | 0.005 |  |  |
| **DQI-I Adequacy Component Scores (0-40)*** | | | | | |
| *Vegetables (0-5)* | 3.0 (3.0) | 3.0 (2.0) | 0.127 |  |  |
| *Fruits (0-5)* | 3.0 (4.0) | 1.0 (5.0) | 0.210 |  |  |
| *Grains (0-5)* | 3.0 (2.0) | 3.0 (2.0) | 0.826 |  |  |
| *Fibre (0-5)* | 5.0 (2.0) | 5.0 (2.0) | 0.367 |  |  |
| *Protein (0-5)* | 5.0 (0.0) | 5.0 (0.0) | 0.624 |  |  |
| *Iron (0-5)* | 5.0 (2.0) | 3.0 (4.0) | 0.042 |  |  |
| *Calcium (0-5)* | 5.0 (3.0) | 5.0 (2.0) | 0.677 |  |  |
| *Vitamin C (0-5)* | 5.0 (1.0) | 3.0 (4.0) | 0.076 |  |  |
| **DQI-I Moderation Component Scores (0-30)*** | | | | | |
| *Total Fat (0-6)* | 0.0 (0.0) | 0.0 (0.0) | 0.709 |  |  |
| *Saturated Fat (0-6)* | 0.0 (0.0) | 0.0 (0.0) | 0.174 |  |  |
| *Cholesterol (0-6)* | 3.0 (6.0) | 6.0 (3.0) | 0.183 |  |  |
| *Sodium (0-6)* | 3.0 (3.0) | 3.0 (6.0) | 0.541 |  |  |
| *Empty Calorie (0-6)* | 0.0 (0.0) | 0.0 (0.0) | 0.941 |  |  |
| **DQI-I Overall Balance Scores (0-10)*** | | | |  |  |
| *Macronutrient Ratio (0-6)* | 0.0 (0.0) | 0.0 (0.0) | 0.388 |  |  |
| *Fatty Acid Ratio (0-4)* | 0.0 (2.0) | 0.0 (0.0) | 0.294 |  |  |
| Parametric variables are presented as mean **±** SD and non-parametric variables as median (interquartile range).  *P*-values were derived with independent sample t-tests (Non-parametric: Mann Whitney U-test).  Significance was derived from a *p*-value of <0.05.  *Non-parametric test.  Of participants who completed the three-day food diary, *n* = 1 did not report whether they used modulators.  Abbreviations: DQI-I, Diet Quality Index – International; SD, standard deviation. | | | | | |
