# Supplementary material for: Interrelationships between diet quality and health-related quality of life in Irish adults living with cystic fibrosis
Source: Eur J Nutr. 2025 Jul 24;64(6):248. doi: 10.1007/s00394-025-03766-y (PMC12289792; doi:10.1007/s00394-025-03766-y)
Supplement: Supplementary file 3 — Supplementary Material 3 [file 394_2025_3766_MOESM3_ESM.docx]

Table S7. Example of large linear regression model completed inclusive of demographic, clinical and PROM variables where the HEI-2020 is the dependent variable.

|  | **B** | **Std. Error** | **β** | ***t*** | ***p*** | **95.0% Confidence Interval for B** | |
| --- | --- | --- | --- | --- | --- | --- | --- |
|  |  |  |  |  |  | **Lower Bound** | **Upper Bound** |
| Model 1 |  |  |  |  |  |  |  |
| (Constant) | 28.990 | 12.475 |  | 2.324 | 0.024 | 3.968 | 54.013 |
| Sex/Gender | 0.349 | 2.699 | 0.019 | 0.129 | 0.898 | -5.064 | 5.762 |
| Age | 0.156 | 0.137 | 0.170 | 1.139 | 0.260 | -0.118 | 0.429 |
| Body Mass Index | 0.370 | 0.426 | 0.161 | 0.868 | 0.389 | -0.485 | 1.225 |
| FEV_1_% | 0.148 | 0.051 | 0.388 | 2.878 | 0.006 | 0.045 | 0.251 |
| Modulator Use | -3.406 | 3.188 | -0.147 | -1.068 | 0.290 | -9.799 | 2.988 |
| Days since last admission to hospital | 0.000 | 0.000 | -0.009 | -0.069 | 0.945 | 0.000 | 0.000 |
| Pancreatic Insufficiency | 0.985 | 3.099 | 0.044 | 0.318 | 0.752 | -5.232 | 7.201 |
| CF-related Diabetes | -1.519 | 2.822 | -0.070 | -0.538 | 0.593 | -7.180 | 4.142 |
| Anxiety / Depression | 2.315 | 3.572 | 0.088 | 0.648 | 0.520 | -4.849 | 9.479 |
| Gastro-Oesophageal Reflux Disease | -3.081 | 3.164 | -0.136 | -0.974 | 0.335 | -9.427 | 3.266 |
| CF-related Liver Disease | -2.396 | 4.249 | -0.080 | -0.564 | 0.575 | -10.918 | 6.127 |
| CF-related Bone Disease | 0.151 | 4.658 | 0.004 | 0.032 | 0.974 | -9.192 | 9.493 |
| Antibiotics | 1.233 | 2.503 | 0.065 | 0.493 | 0.624 | -3.787 | 6.254 |
| Mucolytics | 3.837 | 3.484 | 0.157 | 1.101 | 0.276 | -3.151 | 10.825 |
| Bronchodilators | 3.245 | 2.763 | 0.167 | 1.174 | 0.246 | -2.298 | 8.788 |
| Steroids | 6.233 | 3.747 | 0.229 | 1.663 | 0.102 | -1.283 | 13.748 |
| Fat-Soluble Vitamins | -5.446 | 3.391 | -0.208 | -1.606 | 0.114 | -12.248 | 1.356 |
| Nutritional Supplement Drinks | 5.846 | 3.806 | 0.215 | 1.536 | 0.130 | -1.787 | 13.480 |
| Body Mass Index Categories | -1.931 | 3.806 | -0.100 | -0.507 | 0.614 | -9.566 | 5.704 |
| Model 2 |  |  |  |  |  |  |  |
| (Constant) | 29.776 | 32.831 |  | 0.907 | 0.370 | -36.688 | 96.239 |
| Sex/Gender | 1.110 | 2.868 | 0.059 | 0.387 | 0.701 | -4.695 | 6.915 |
| Age | 0.295 | 0.161 | 0.322 | 1.837 | 0.074 | -0.030 | 0.620 |
| Body Mass Index | 0.191 | 0.468 | 0.083 | 0.408 | 0.686 | -0.757 | 1.139 |
| FEV_1_% | 0.152 | 0.063 | 0.398 | 2.393 | 0.022 | 0.023 | 0.280 |
| Modulator Use | -0.276 | 3.413 | -0.012 | -0.081 | 0.936 | -7.186 | 6.633 |
| Days since last admission to hospital | 0.000 | 0.000 | 0.078 | 0.493 | 0.625 | 0.000 | 0.001 |
| Pancreatic Insufficiency | 3.184 | 3.424 | 0.144 | 0.930 | 0.358 | -3.748 | 10.115 |
| CF-related Diabetes | -0.042 | 3.687 | -0.002 | -0.011 | 0.991 | -7.505 | 7.422 |
| Anxiety / Depression | 5.257 | 4.740 | 0.201 | 1.109 | 0.274 | -4.338 | 14.853 |
| Gastro-Oesophageal Reflux Disease | 0.394 | 3.886 | 0.017 | 0.101 | 0.920 | -7.474 | 8.262 |
| CF-related Liver Disease | -2.377 | 5.319 | -0.079 | -0.447 | 0.657 | -13.145 | 8.391 |
| CF-related Bone Disease | 1.563 | 5.243 | 0.046 | 0.298 | 0.767 | -9.051 | 12.177 |
| Antibiotics | -1.660 | 2.979 | -0.088 | -0.557 | 0.581 | -7.692 | 4.371 |
| Mucolytics | 2.599 | 3.799 | 0.106 | 0.684 | 0.498 | -5.091 | 10.289 |
| Bronchodilators | 2.648 | 3.080 | 0.136 | 0.860 | 0.395 | -3.587 | 8.883 |
| Steroids | 6.940 | 4.286 | 0.255 | 1.619 | 0.114 | -1.736 | 15.615 |
| Fat-Soluble Vitamins | -7.600 | 3.843 | -0.290 | -1.978 | 0.055 | -15.379 | 0.180 |
| Nutritional Supplement Drinks | 5.614 | 4.323 | 0.206 | 1.299 | 0.202 | -3.137 | 14.365 |
| Body Mass Index Categories | 1.502 | 4.438 | 0.078 | 0.338 | 0.737 | -7.483 | 10.487 |
| Physical Functioning - CFQ-R score | 0.119 | 0.109 | 0.305 | 1.089 | 0.283 | -0.102 | 0.339 |
| Vitality - CFQ-R score | -0.104 | 0.109 | -0.212 | -0.952 | 0.347 | -0.326 | 0.117 |
| Emotional Functioning - CFQ-R score | 0.169 | 0.111 | 0.340 | 1.530 | 0.134 | -0.055 | 0.393 |
| Eating Problems - CFQ-R score | -0.087 | 0.202 | -0.117 | -0.430 | 0.669 | -0.496 | 0.322 |
| Treatment Burden - CFQ-R score | -0.014 | 0.088 | -0.032 | -0.162 | 0.872 | -0.192 | 0.163 |
| Social/School Functioning - CFQ-R score | 0.082 | 0.080 | 0.167 | 1.021 | 0.314 | -0.080 | 0.244 |
| Body Image - CFQ-R score | 0.070 | 0.072 | 0.179 | 0.973 | 0.336 | -0.076 | 0.216 |
| Role Functioning - CFQ-R score | -0.091 | 0.136 | -0.160 | -0.670 | 0.507 | -0.366 | 0.184 |
| Weight - CFQ-R score | -0.126 | 0.079 | -0.323 | -1.606 | 0.117 | -0.285 | 0.033 |
| Respiratory Symptoms - CFQ-R score | -0.075 | 0.133 | -0.099 | -0.567 | 0.574 | -0.344 | 0.193 |
| Self-reported health scale score - EQ-5D-5L | -0.020 | 0.145 | -0.030 | -0.137 | 0.892 | -0.314 | 0.275 |
| Mobility (Walking) - EQ-5D-5L | 0.625 | 4.600 | 0.036 | 0.136 | 0.893 | -8.688 | 9.937 |
| EQ-5D-5L Index value (Irish) | -12.913 | 8.746 | -0.222 | -1.476 | 0.148 | -30.618 | 4.792 |
| PAGI-SYM score | -0.777 | 4.513 | -0.047 | -0.172 | 0.864 | -9.912 | 8.358 |
| Digestive Symptoms - CFQ-R score | 0.192 | 0.083 | 0.414 | 2.313 | 0.026 | 0.024 | 0.361 |
| Dependent Variable: HEI Score. *p* = 0.006, R2adj = 0.677 | | | | | | | |
|  | | | | | | | |

Table S8. Example of large linear regression model completed inclusive of demographic, clinical and PROM variables where the DQI-I is the dependent variable.

|  | **B** | **Std. Error** | **β** | ***t*** | ***p*** | **95% Confidence Interval for B** | |
| --- | --- | --- | --- | --- | --- | --- | --- |
|  |  |  |  |  |  | **Lower bound** | **Upper Bound** |
| Model 1 |  |  |  |  |  |  |  |
| (Constant) | 28.990 | 12.475 |  | 2.324 | 0.024 | 3.968 | 54.013 |
| Sex/Gender | 0.349 | 2.699 | 0.019 | 0.129 | 0.898 | -5.064 | 5.762 |
| Age | 0.156 | 0.137 | 0.170 | 1.139 | 0.260 | -0.118 | 0.429 |
| Body Mass Index | 0.370 | 0.426 | 0.161 | 0.868 | 0.389 | -0.485 | 1.225 |
| FEV_1_% | 0.148 | 0.051 | 0.388 | 2.878 | 0.006 | 0.045 | 0.251 |
| Modulator Use | -3.406 | 3.188 | -0.147 | -1.068 | 0.290 | -9.799 | 2.988 |
| Days since last admission to hospital | 0.000 | 0.000 | -0.009 | -0.069 | 0.945 | 0.000 | 0.000 |
| Pancreatic Insufficiency | 0.985 | 3.099 | 0.044 | 0.318 | 0.752 | -5.232 | 7.201 |
| CF-related Diabetes | -1.519 | 2.822 | -0.070 | -0.538 | 0.593 | -7.180 | 4.142 |
| Anxiety / Depression | 2.315 | 3.572 | 0.088 | 0.648 | 0.520 | -4.849 | 9.479 |
| Gastro-Oesophageal Reflux Disease | -3.081 | 3.164 | -0.136 | -0.974 | 0.335 | -9.427 | 3.266 |
| CF-related Liver Disease | -2.396 | 4.249 | -0.080 | -0.564 | 0.575 | -10.918 | 6.127 |
| CF-related Bone Disease | 0.151 | 4.658 | 0.004 | 0.032 | 0.974 | -9.192 | 9.493 |
| Antibiotics | 1.233 | 2.503 | 0.065 | 0.493 | 0.624 | -3.787 | 6.254 |
| Mucolytics | 3.837 | 3.484 | 0.157 | 1.101 | 0.276 | -3.151 | 10.825 |
| Bronchodilators | 3.245 | 2.763 | 0.167 | 1.174 | 0.246 | -2.298 | 8.788 |
| Steroids | 6.233 | 3.747 | 0.229 | 1.663 | 0.102 | -1.283 | 13.748 |
| Fat-Soluble Vitamins | -5.446 | 3.391 | -0.208 | -1.606 | 0.114 | -12.248 | 1.356 |
| Nutritional Supplement Drinks | 5.846 | 3.806 | 0.215 | 1.536 | 0.130 | -1.787 | 13.480 |
| Body Mass Index Categories | -1.931 | 3.806 | -0.100 | -0.507 | 0.614 | -9.566 | 5.704 |
| Model 2 |  |  |  |  |  |  |  |
| (Constant) | 29.776 | 32.831 |  | 0.907 | 0.370 | -36.688 | 96.239 |
| Sex/Gender | 1.110 | 2.868 | 0.059 | 0.387 | 0.701 | -4.695 | 6.915 |
| Age | 0.295 | 0.161 | 0.322 | 1.837 | 0.074 | -0.030 | 0.620 |
| Body Mass Index | 0.191 | 0.468 | 0.083 | 0.408 | 0.686 | -0.757 | 1.139 |
| FEV_1_% | 0.152 | 0.063 | 0.398 | 2.393 | 0.022 | 0.023 | 0.280 |
| Modulator Use | -0.276 | 3.413 | -0.012 | -0.081 | 0.936 | -7.186 | 6.633 |
| Days since last admission to hospital | 0.000 | 0.000 | 0.078 | 0.493 | 0.625 | 0.000 | 0.001 |
| Pancreatic Insufficiency | 3.184 | 3.424 | 0.144 | 0.930 | 0.358 | -3.748 | 10.115 |
| CF-related Diabetes | -0.042 | 3.687 | -0.002 | -0.011 | 0.991 | -7.505 | 7.422 |
| Anxiety / Depression | 5.257 | 4.740 | 0.201 | 1.109 | 0.274 | -4.338 | 14.853 |
| Gastro-Oesophageal Reflux Disease | 0.394 | 3.886 | 0.017 | 0.101 | 0.920 | -7.474 | 8.262 |
| CF-related Liver Disease | -2.377 | 5.319 | -0.079 | -0.447 | 0.657 | -13.145 | 8.391 |
| CF-related Bone Disease | 1.563 | 5.243 | 0.046 | 0.298 | 0.767 | -9.051 | 12.177 |
| Antibiotics | -1.660 | 2.979 | -0.088 | -0.557 | 0.581 | -7.692 | 4.371 |
| Mucolytics | 2.599 | 3.799 | 0.106 | 0.684 | 0.498 | -5.091 | 10.289 |
| Bronchodilators | 2.648 | 3.080 | 0.136 | 0.860 | 0.395 | -3.587 | 8.883 |
| Steroids | 6.940 | 4.286 | 0.255 | 1.619 | 0.114 | -1.736 | 15.615 |
| Fat-Soluble Vitamins | -7.600 | 3.843 | -0.290 | -1.978 | 0.055 | -15.379 | 0.180 |
| Nutritional Supplement Drinks | 5.614 | 4.323 | 0.206 | 1.299 | 0.202 | -3.137 | 14.365 |
| Body Mass Index Categories | 1.502 | 4.438 | 0.078 | 0.338 | 0.737 | -7.483 | 10.487 |
| Physical Functioning - CFQ-R score | 0.119 | 0.109 | 0.305 | 1.089 | 0.283 | -0.102 | 0.339 |
| Vitality - CFQ-R score | -0.104 | 0.109 | -0.212 | -0.952 | 0.347 | -0.326 | 0.117 |
| Emotional Functioning - CFQ-R score | 0.169 | 0.111 | 0.340 | 1.530 | 0.134 | -0.055 | 0.393 |
| Eating Problems - CFQ-R score | -0.087 | 0.202 | -0.117 | -0.430 | 0.669 | -0.496 | 0.322 |
| Treatment Burden - CFQ-R score | -0.014 | 0.088 | -0.032 | -0.162 | 0.872 | -0.192 | 0.163 |
| Social/School Functioning - CFQ-R score | 0.082 | 0.080 | 0.167 | 1.021 | 0.314 | -0.080 | 0.244 |
| Body Image - CFQ-R score | 0.070 | 0.072 | 0.179 | 0.973 | 0.336 | -0.076 | 0.216 |
| Role Functioning - CFQ-R score | -0.091 | 0.136 | -0.160 | -0.670 | 0.507 | -0.366 | 0.184 |
| Weight - CFQ-R score | -0.126 | 0.079 | -0.323 | -1.606 | 0.117 | -0.285 | 0.033 |
| Respiratory Symptoms - CFQ-R score | -0.075 | 0.133 | -0.099 | -0.567 | 0.574 | -0.344 | 0.193 |
| Self-reported health scale score - EQ-5D-5L | -0.020 | 0.145 | -0.030 | -0.137 | 0.892 | -0.314 | 0.275 |
| Mobility (Walking) - EQ-5D-5L | 0.625 | 4.600 | 0.036 | 0.136 | 0.893 | -8.688 | 9.937 |
| EQ-5D-5L Index value (Irish) | -12.913 | 8.746 | -0.222 | -1.476 | 0.148 | -30.618 | 4.792 |
| PAGI-SYM score | -0.777 | 4.513 | -0.047 | -0.172 | 0.864 | -9.912 | 8.358 |
| Digestive Symptoms - CFQ-R score | 0.192 | 0.083 | 0.414 | 2.313 | 0.026 | 0.024 | 0.361 |
| Dependent Variable: DQI-I Score. *p* = 0.133 | | | | | | | |
